# Comparative study on anatomical traits and gas exchange responses due to belowground hypoxic stress and thermal stress in three tropical seagrasses

**DOI:** 10.7717/peerj.12899

**Published:** 2022-02-09

**Authors:** Sutthinut Soonthornkalump, Yan Xiang Ow, Chanida Saewong, Pimchanok Buapet

**Affiliations:** 1 Division of Biological Science, Faculty of Science, Prince of Songkla University, Hat Yai, Songkhla, Thailand; 2 Coastal Oceanography and Climate Change Research Center, Prince of Songkla University, Hat Yai, Songkhla, Thailand; 3 St John’s Island National Marine Laboratory, Tropical Marine Science Institute, National University of Singapore, Singapore, Singapore

**Keywords:** Seagrasses, Anatomy, Gas exchange, Hypoxia, Thermal stress

## Abstract

**Background:**

The ability to maintain sufficient oxygen levels in the belowground tissues and the rhizosphere is crucial for the growth and survival of seagrasses in habitats with highly reduced sediment. Such ability varies depending on plant anatomical features and environmental conditions.

**Methods:**

In the present study, we compared anatomical structures of roots, rhizomes and leaves of the tropical intertidal seagrasses, *Cymodocea rotundata*, *Thalassia hemprichii* and *Halophila ovalis*, followed by an investigation of their gas exchange both in the belowground and aboveground tissues and photosynthetic electron transport rates (ETR) in response to experimental manipulations of O_2_ level (normoxia and root hypoxia) and temperature (30 °C and 40 °C).

**Results:**

We found that *C. rotundata* and *T. hemprichii* displayed mostly comparable anatomical structures, whereas *H. ovalis* displayed various distinctive features, including leaf porosity, number and size of lacunae in roots and rhizomes and structure of radial O_2_ loss (ROL) barrier. *H. ovalis* also showed unique responses to root hypoxia and heat stress. Root hypoxia increased O_2_ release from belowground tissues and overall photosynthetic activity of *H. ovalis* but did not affect the other two seagrasses. More pronounced warming effects were detected in *H. ovalis*, measured as lower O_2_ release in the belowground tissues and overall photosynthetic capacity (O_2_ release and dissolved inorganic carbon uptake in the light and ETR). High temperature inhibited photosynthesis of *C. rotundata* and *T. hemprichii* but did not affect their O_2_ release in belowground tissues. Our data show that seagrasses inhabiting the same area respond differently to root hypoxia and temperature, possibly due to their differences in anatomical and physiological attributes. *Halophila ovalis* is highly dependent on photosynthesis and appears to be the most sensitive species with the highest tendency of O_2_ loss in hypoxic sediment. At the same time, its root oxidation capacity may be compromised under warming scenarios.

## Introduction

Successful colonization of seagrasses in coastal habitats requires multi-faceted adaptations encompassing molecular, physiological, anatomical and morphological levels (Kiswara et al., 2009; Papenbrock, 2012; Brodersen et al., 2018a; Olsen et al., 2018). In addition to characteristics acquired to live underwater (Papenbrock, 2012; Olsen et al., 2016), the ability to cope with highly reduced and oxygen-deficient sediment is among the most crucial traits of seagrasses (Brodersen et al., 2018a, 2018b; Olsen et al., 2018; Martin et al., 2019; Zhang et al., 2021).

Seagrasses have developed the aerenchyma system in mesophylls, rhizomes and roots cortex, giving these tissues relatively high porosity (Brodersen et al., 2018a; Martin et al., 2019). This aerenchyma network provides low resistance for oxygen transport from the leaves to the roots (Kuo & den Hartog, 2006; Steffens, Geske & Sauter, 2011; Novak & Short, 2014; Brodersen et al., 2018a, 2018b; Martin et al., 2019). This oxygen supply not only maintains root cellular respiration in anoxic sediment (Connell, Colmer & Walker, 1999; Pezeshki & DeLaune, 2012), it also creates an oxygenated microenvironment in the rhizosphere through radial oxygen loss (ROL), vital for preventing toxicity of sulphide, a predominant phytotoxin in seagrass sediments (Brodersen et al., 2016, 2018b; Martin et al., 2019). A balance between oxygen supply and oxygen loss in the belowground tissue needs to be regulated as high ROL might lead to oxygen shortage in the root apex (Martin et al., 2019). Anatomical structure acting as a ROL barrier observed as an accumulation of suberin or lignin at the middle lamella of subepidermal layers termed exodermis has been identified in the seagrasses, *Halophila ovalis*, *Cymodocea rotundata* and *Zostera marina* (Pedersen et al., 1998; Connell, Colmer & Walker, 1999; Holmer & Bondgaard, 2001; Sand-Jensen et al., 2005). These aerenchyma networks and ROL barriers have also been found in other wetland plants (Armstrong, 1971; Pi et al., 2009; Lemoine et al., 2012; Collier & Waycott, 2014; Chorchuhirun, Kraichak & Kermanee, 2020; Ejiri, Sawazaki & Shiono, 2020; Tatongjai, Kraichak & Kermanee, 2021) and certain crop species, such as taro and rice (Abiko & Miyasaka, 2020; Mei et al., 2020).

These anatomical characteristics are tightly coupled to fundamental physiological functions of plants, such as cellular respiration and photosynthesis. Their roles in mediating plant stress tolerance have been extensively explored in many plant species (Jensen et al., 2005; Pi et al., 2009; Jovanovic et al., 2015; Dai et al., 2017; Cheng et al., 2020a, 2020b; de Lopes et al., 2020; Mei et al., 2020; Scholz et al., 2021). Studies in mangrove plants suggest that differences in both porosity and ROL barrier contribute to different sensitivity to waterlogging, root zone hypoxia, nutrient limitation and heavy metal stress (Pi et al., 2009; Dai et al., 2017; Cheng et al., 2020a, 2020b; Pedersen et al., 2021). In-depth studies of the rhizosphere environments in seagrasses thus far have focused on temperate species, such as *Zostera marina*, *Z. muelleri, Ruppia maritima* and *Posidonia australis* (Brodersen et al., 2015a, 2015b, 2016, 2017a, 2018a, 2018b; Martin et al., 2019). While the functional relationship between anatomical traits and gas exchange under changing environments is less frequently examined in tropical seagrasses, field investigations have shown that the sediment biogeochemistry in the tropical seagrass meadow is driven by the photosynthetic activity of seagrasses *(*Brodersen et al., 2017b; Martin et al., 2018; Schrameyer et al., 2018; Lyimo et al., 2018), highlighting the connection between the photosynthetic and the belowground tissues, possibly by means of gas exchange and transport, as well as exudation of carbon and nitrogen.

Increasing temperature affects seagrass energy balance and primary production (George et al., 2018; Kong et al., 2019; Rasmusson et al., 2020, 2021). Both photosynthesis and respiration are controlled by temperature; however, photosynthesis often shows lower optimal temperature than respiration (Marín-Guirao et al., 2016; Pedersen et al., 2016; George et al., 2018; Rasmusson et al., 2020, 2021). Since the ability to sustain oxygen in the root tissues and the rhizosphere is determined by, but not limited to, photosynthetic oxygen production and respiratory oxygen consumption, the increasing temperature may affect seagrass species differently, depending on their sensitivity to warming of photosynthesis and respiration and their relevant anatomical features such as tissue porosity and barrier to ROL. Low oxygen levels in meristematic tissues and heat stress have been proposed to cause seagrass die-off in many locations (Greve, Borum & Pedersen, 2003; Borum et al., 2005; Carlson et al., 2018; Strydom et al., 2020). In addition, tropical seagrasses, living close to their thermal threshold, are particularly vulnerable to warming (Rasmusson et al., 2020, 2021). These observations indicate a pressing need for a better mechanistic understanding of the capability of tropical seagrasses to cope with hypoxia and heat stress.

The seagrasses, *Cymodocea rotundata, Thalassia hemprichii* and *Halophila ovalis* are commonly found in shallow coastal areas in the tropical Southeast Asian region (Buapet, Makkliang & Thammakhet-Buranachai, 2017; Phandee & Buapet, 2018; Kong et al., 2019; Ow et al., 2020). In such locations, they are often disturbed by human activities, which subsequently reduce their photosynthetic capacity (Yaakub et al., 2014; Browne et al., 2017) and/or induce localized hypoxia in the water column and sediment (Altieri et al., 2021) while being exposed to high temperatures at midday and during low tide (Pedersen et al., 2016; George et al., 2018; Rasmusson et al., 2020). In Phuket, Thailand, these three seagrasses co-occur in the shallow soft-bottom habitat, making them relevant subjects for a comparative study.

In the present study, we investigated anatomical characteristics of *C. rotundata*, *T. hemprichii* and *H. ovalis* and their gas exchange and photosynthetic responses to root hypoxia and heat stress. Oxygen and dissolved inorganic carbon (DIC) exchange rates of the belowground and aboveground tissues and the electron transport rates of photosystem II were measured and subsequently linked to differences in anatomical structures. We aim to improve an understanding of seagrass functional anatomy and its role in stress tolerance to belowground hypoxia and high temperature. The results obtained in this study also provide insight into differing sensitivity to root hypoxia and high temperature in tropical seagrasses.

## Materials and Methods

### Plant materials and preparation

In July 2021, intact plants of three seagrass species: *Cymodocea rotundata* Asch. & Schweinf., *Thalassia hemprichii* (Ehrenb.) Asch. and *Halophila ovalis* (R.Br.) Hook.f. were collected from the intertidal seagrass bed at Pakhlok subdistrict, Phuket, Thailand (8°01′18.3″N 98°24′47.2″E). The collected seagrasses were kept in a storage tank containing aerated artificial seawater (ASW; 30 ppt, pH 8.1 ± 0.3), at 29 ± 1 °C under 12/12 h photoperiod (200 µmol m^−2^s^−1^). The conditions of the storage tank mimicked the environmental conditions in the natural setting: salinity was chosen based on the salinity of the seawater at the collection site, whereas temperature and irradiance represent daily average temperature and irradiance within the month of sampling, respectively. The ASW was prepared by dissolving synthetic sea salt (Mariscience International, Thailand) in deionized water until the salinity of 30 (practical salinity unit, PSU) was reached and kept aerated overnight prior to use. The collected seagrass specimens with healthy roots and intact leaves were rinsed with ASW to clean off the sediment and epiphytes. Samplings for anatomical analyses were done immediately. Afterwards, the horizontal rhizomes were separated into individual ramets by excision with a razor blade. Our investigation solely focused on the youngest mature ramets as they represent a significant site with a fully developed structure and function. These youngest mature ramets are metabolically active, and they have been widely used in physiological investigations (Short & Duarte, 2001; Collier et al., 2008; Park et al., 2016; Schubert et al., 2018; Rasmusson et al., 2019; Nguyen et al., 2020). Following the literature mentioned above, we used the third ramet from the growing apex of *C. rotundata* and *T. hemprichii* and the second ramet from the growing apex of *H. ovalis*. The excised seagrasses were acclimatized in the above condition of ASW for 1 day before use in the experiment.

### Characterization of the anatomical features and localization of oxygen loss in intact roots

**Figure 1:**
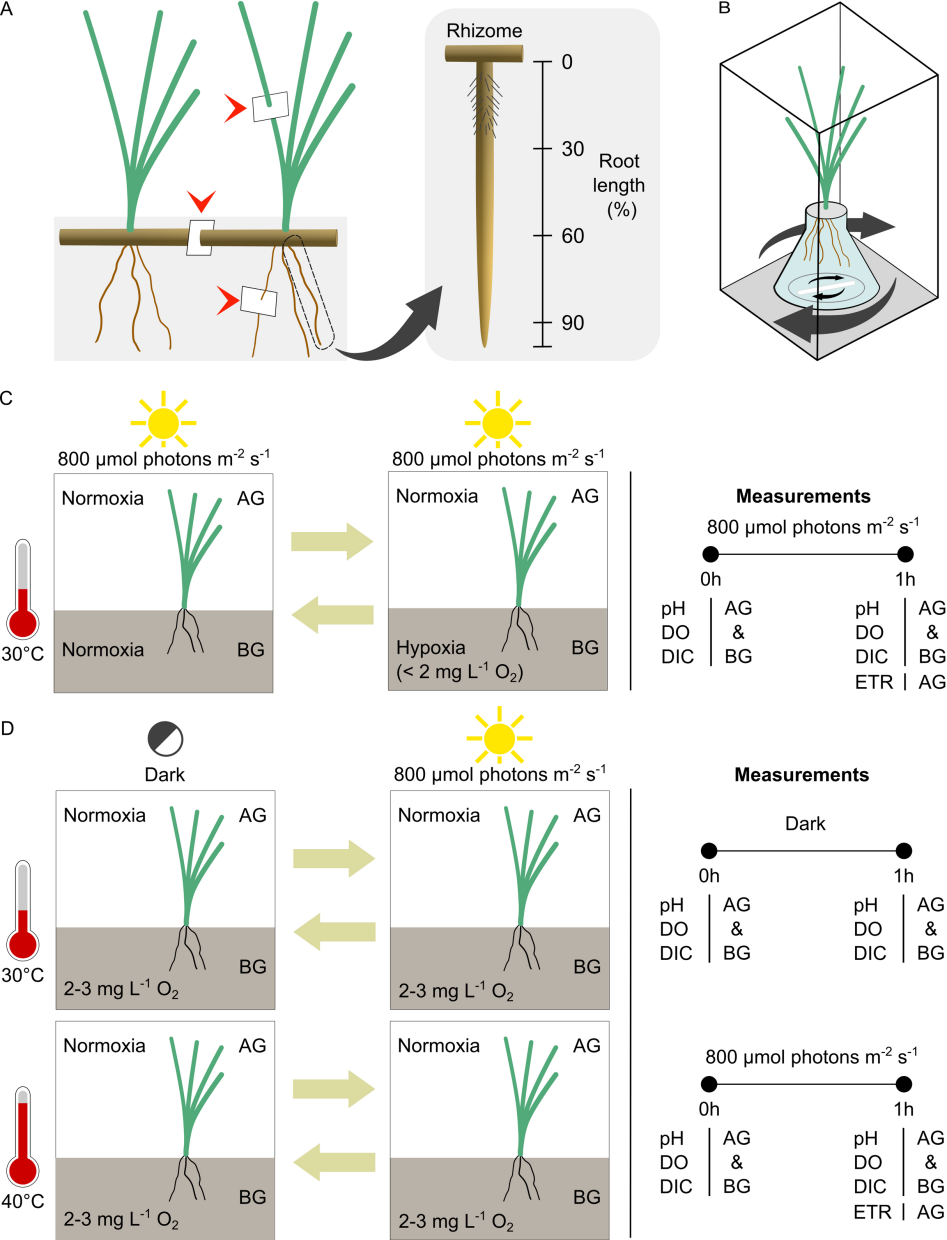
Experimental setup and design. (A) Location of leaf, rhizome and root tissue sampling for anatomical study (arrow heads) and the different root zones examined in the root porosity assessment. (B) The incubation chamber setup. (C) The experimental design for comparison of seagrass gas exchange and electron transport rates in contrasting oxygen levels in the belowground compartment. (D) The experimental design for comparison of seagrass gas exchange and electron transport rates at optimal and high temperatures. AG, aboveground compartment. BG, belowground compartment.

The youngest mature ramets of *C. rotundata*, *T. hemprichii* and *H. ovalis* were used for this investigation. For the leaves and roots, the sampling position was at the middle area of the total length (Fig. 1A). The rhizome tissues were collected at the mid-length of the internodes between the second and the third shoots (Fig. 1A). The collected tissues were fixed in FAA II (90% ethanol, 5% formaldehyde, 5% glacial acetic acid) for 48 h (Ruzin, 1999). The conventional paraffin method was used (Johansen, 1940). Fixed specimens were dehydrated by tertiary-butyl-alcohol series (70%, 85%, 95% and 100%), each for 24 h, respectively. Then a mixture of paraffin oil and paraffin wax (1:1 v/v) was used for infiltration, followed by three changes of paraffin wax in a hot air oven (56 °C), each for 24 h. Specimens were embedded in paraffin wax and cut at 20–30 µm in thickness with a rotary microtome (Shandon AS325; Fisher scientific, Hampton, NH, USA). Sections were affixed on the cleaned glass slide. The simple toluidine blue O staining method was used in histological observation (Sakai, 1973). The affixed sections were stained with 0.05% toluidine blue O for 5–10 min. Then, they were rinsed in distilled water for 1 min and dried in the air-flow cabinet. Stained sections were deparaffinized with two changes of xylene and mounted on the slide with Permount™ (Fisher, Hampton, NH, USA). A lignified tissue, suberized tissue and tannins showed blue-green colour while red-purple colour was displayed in non-lignified structures. Stained sections were observed using a light microscope (BX 51 TRF; Olympus; Tokyo, Japan) and photographed by a digital camera (DP72; Olympus; Tokyo, Japan). Anatomical features of leaf, root, and rhizome were measured from three individual plants per species; each replicate measurement consisted of three sub-replicate measurements of the same plant.

Root porosity was measured by cross-section analysis (Visser & Bögemann, 2003). The whole root was divided into three zones, basal, middle, and apical (0–30%, 30–60%, and 60–90% of root length measured from a rhizome, respectively) (Fig. 1A). Root samples were collected from each zone by transverse dissection and stained with 0.05% toluidine blue O for 5–10 min. The sections were observed under a light compound microscope. The area occupied by air space and total cross-section area was measured using ImageJ software (U. S. National Institutes of Health, Bethesda, MD, USA). Root tissues were sampled from 5 individual plants of each species. The porosity was estimated from the equation below.

$${\rm{Porosity}} = {{area\>of\>air\>space} \over {{\rm{total}}\>{\rm{cross}} - {\rm{section}}\>{\rm{area}}}}.100$$

Localization of oxygen loss in intact roots of the youngest mature ramets was done *via* staining due to the oxidation of methylene blue (Abiko & Miyasaka, 2020). The working solution of methylene blue was freshly prepared by dissolving 13 mg (0.041 mmol) methylene blue into 1 L of ASW. Then, the Sodium dithionite (Na_2_S_2_O_4_) was weighted at 130 mg L^−1^ (0.075 mmol) and added into the methylene blue solution. This step reduced the oxidized dye, and the working solution became colourless. Seagrasses belowground tissues were submerged into a 250 mL Erlenmeyer flask containing the reaction solution. The setup was firmly wrapped with parafilm and carefully placed in a 500 mL beaker containing ASW. Staining was performed for 60 min in light condition (800 μmol photons m^−2^s^−1^) at 30 °C. After 60 min of staining, seagrass was transferred in the rectangular acrylic chamber (6.5 × 6.5 × 19 cm) containing ASW to stop the reaction and photographed using a digital camera (SX50 HS; Canon; Tokyo, Japan). The tissue stained in blue was where oxidation (by released oxygen) occurred, while colourless tissue indicated a reducing area.

### Experimental design and setup

The incubation chamber system (Fig. 1B) was modified from Peyer, Maricle & Young (2020). It consists of aboveground (AG) and belowground (BG) compartments. The youngest mature ramets of the same species from different sods were randomly bundled together (five ramets for *C. rotundata*, two ramets for *T. hemprichii* and 15 ramets for *H. ovalis*). The seagrass below ground tissues were incubated in a 25 mL Erlenmeyer flask (Corning, MA, USA). The bases of the shoots were firmly wrapped using parafilm (Bemis, Neenah, WI, USA) and secured to the opening of the Erlenmeyer flask using plasticine. Each seagrass bundle was individually placed into a rectangular transparent acrylic chamber (6.5 × 6.5 × 19 cm; 802.75 mL for *C. rotundata* and *T. hemprichii* and 6.5 × 6.5 × 15 cm; 633.75 mL for *H. ovalis*) filled with ASW. A magnetic stir bar was included in both the BG and AG compartment to ensure the homogenous flow of ASW throughout the incubation. The light was provided using aquarium LED (Chichiros; Shanghai Ogino Biotechnology, Shanghai, China). The temperature was controlled using a hotplate stirrer (M TOPS; Misung Scientific, Korea).

### Comparing seagrass gas exchange and photosynthetic electron transport rates in contrasting oxygen levels in the belowground compartment

The equipment was prepared as described above. This experiment aimed to compare gas exchange in the light in the belowground (BG) and aboveground (AG) compartments in two conditions (1) Normoxia: both BG and AG compartments had initial dissolved oxygen (DO) approx. 6–7 mg L^−1^ and (2) Root hypoxia: BG compartments had initial dissolved oxygen (DO) ≤ 2 mg L^−1,^ and AG compartments had initial dissolved oxygen (DO) approx. 6–7 mg L^−1^. The DO levels were chosen based on the values recorded *in-situ* (2–6 mg L^−1^ for porewater DO and 6–8 mg L^−1^ for water column DO) and a range of DO in tropical seagrass meadows reported in previous studies (Erftemeijer, Osinga & Mars, 1993; Satumanatpan, Thummikkapong & Kanongdate, 2011; Ahmad-Kamil et al., 2013; Shi et al., 2015; Singh et al., 2015; Ganguly et al., 2017). The DO of ASW was manipulated by purging with N_2_ gas at a flow rate of 25 mL S^−1^. It is worth noting that seawater pH increased by 0.3 units (from approximately 8.1 to 8.4) while purging with N_2_ gas in the root hypoxia treatments. However, as DO manipulation was only applied to belowground tissue, pH changes should not affect photosynthetic activity. We used ASW instead of natural seawater to minimize interference from microbial activity. Nevertheless, the control chamber containing ASW without seagrass was included in every round of measurement.

A reciprocal approach was adopted to examine the effects of root hypoxia using different orders; the first setup began with normoxia for 1 h followed by root hypoxia for 1 h, whereas the second setup began with root hypoxia for 1 h followed by normoxia for 1 h (Fig. 1C). The order was randomly assigned to each run so that six replicate runs were done using one order, and the other six were run using the opposite order. By including both orders, we could examine the effects of belowground hypoxia while integrating differences that may have occurred due to stress effects of incubation time. The ASW was renewed each time the DO was changed. The temperature was maintained at 30 °C. The light was set at 800 µmol photons m^−2^ s^−1^ (the minimal irradiance in which the BG shows detectable net oxygen release in our experimental setup, pre-determined in the pilot study).

The DO, pH and total alkalinity (TA) of the ASW were measured before and after incubation in each condition. The DO and pH were measured using Multiparameter benchtop meter inoLab® Multi 9630 IDS (Xylem Analytics, Oberbayern, Germany). The multiparameter benchtop meter operates within a pH range of 0–14 and temperature of 0–80 °C. Salinity correction was done during calibration. Both DO sensors and pH electrodes are equipped with integrated temperature sensors, and the values provided in the readings are temperature-compensated. The TA was determined using the rapid titration method following Buapet, Gullström & Björk (2013). At the end of the experiment, the seagrass specimens were separated into AG and BG tissues and the dry weight was assessed after drying in a hot air oven at 70 °C for 72 h. The net oxygen release/uptake rates were calculated from a difference between initial and final DO and normalized to the dry weight (DW) of seagrass material and the incubation duration. The dissolved inorganic carbon (DIC) concentration was calculated following the method described by Buapet, Gullström & Björk (2013). The net DIC release/uptake rates were calculated from a difference between initial and final DIC and normalized to the dry weight (DW) of seagrass material and the incubation duration. The change detected in the negative control (plant-free setup) was deducted from the analysis before calculating oxygen and DIC exchange rates. Each treatment contained 12 replications.

Photosynthetic activity was determined at the end of the light exposure in each condition as the electron transport rates (ETR) from photosystem II. The ETR was calculated by multiplying the effective quantum yield (φPSII) by the irradiance (800 μmol photons m^−2^ s^−1^) and by 0.5 (equal distribution of photon energy between photosystem I and photosystem II), and by the absorption factor (AF, estimated following the method described by Beer & Björk (2000)).

### Comparing seagrass gas exchange in optimal and warming temperatures

The equipment was prepared as previously described. All incubation chambers had the same initial dissolved oxygen (DO): BG compartments had an initial DO of approx. 2 mg L^−1,^ and AG compartments had an initial DO of approx. 6–7 mg L^−1^. As the temperature is known to influence dark respiration as well as photosynthesis, this experiment aimed to compare gas exchange both in the light and in the dark in the BG and AG compartments in two conditions: optimal temperature (30 °C, pre-determined in the previous experiment) and warming treatment (40 °C, corresponding to the highest temperature recorded *in-situ*).

Two independent temperature treatments were tested with light/dark reciprocal design, (1) the temperature was set at 30 °C, the measurement began with dark incubation for 1 h followed by light incubation for 1 h or the temperature was set at 30 °C, the measurement began with light incubation for 1 h followed by dark incubation for 1 h and (2) the temperature was set at 40 °C, the measurement began with dark incubation for 1 h followed by light incubation for 1 h or the temperature was set at 40 °C, the measurement began light incubation for 1 h followed by dark incubation for 1 h (Fig. 1D). The orders were randomly assigned to each run so that three replicate runs were assessed for each order. Similar to the previous experiment, we adopted a reciprocal approach to avoid interference of the stress effects of incubation time. The control chamber containing ASW without seagrass was included in each treatment. The ASW was renewed each time the light was changed. The light was set at 800 μmol photons m^−2^ s^−1^.

The DO, pH and total alkalinity (TA) were measured before and after each incubation. The DO and pH were measured using Multiparameter benchtop meter inoLab® Multi 9630 IDS (Xylem Analytics, Oberbayern, Germany). The TA was determined using the rapid titration method following Buapet, Gullström & Björk (2013). At the end of the experiment, the seagrass specimens were separated into AG and BG tissues and the dry weight was assessed after drying in a hot air oven at 70 °C for 72 h. Net oxygen release/uptake rates and DIC release/uptake rates were calculated as previously described. The change detected in the negative control (plant-free setup) was deducted from the analysis before calculating oxygen and DIC exchange rates.

In addition, the electron transport rates were determined at the end of the light exposure in each condition as previously described.

### Data analysis

The difference in all the anatomical features among species was analyzed using one-way ANOVA except for root porosity and diameter, in which two-way ANOVA was used. Multiple comparison tests were performed using Duncan’s Multiple Range Test (DMRT).

In the first experimental setup, repeated-measures ANOVA was used to establish differences in the oxygen and dissolved inorganic carbon (DIC) exchange rates in BG and AG tissues among oxygen levels (normoxia vs hypoxia) and species (*Cymodocea rotundata*, *Thalassia hemprichii* and *Halophila ovalis*). Oxygen level was used as a within-group factor, and species were used as categorical factors. Multiple comparison tests were performed using Tukey’s honestly significant difference (HSD) test. The difference in electron transport rates (ETR) was analyzed using repeated-measures ANOVA with oxygen level set as within-group factor and species was set as categorical factors. Multiple comparison tests were performed using Tukey’s HSD post-hoc test.

In the second experimental setup, repeated-measures ANOVA was used to establish differences in the oxygen and dissolved inorganic carbon (DIC) exchange rates in BG tissues among light levels (dark and light), temperature levels (30 °C and 40 °C) and species (*C. rotundata*, *T. hemprichii* and *H. ovalis*). The light level was used as a within-group factor, and temperatures and species were used as categorical factors. Multiple comparison tests were performed using Tukey’s honestly significant difference (HSD) test. For the AG tissues, values obtained in the dark (net oxygen uptake and net DIC release) and values obtained in the light (net oxygen release, net DIC uptake and ETR) were tested separately using two-way ANOVA with temperatures and species set as categorical factors. Multiple comparison tests were performed using Tukey’s honestly significant difference (HSD) test.

## Results

### Anatomical features and localization of the oxygen loss in intact roots

#### Root anatomy

**Figure 2:**
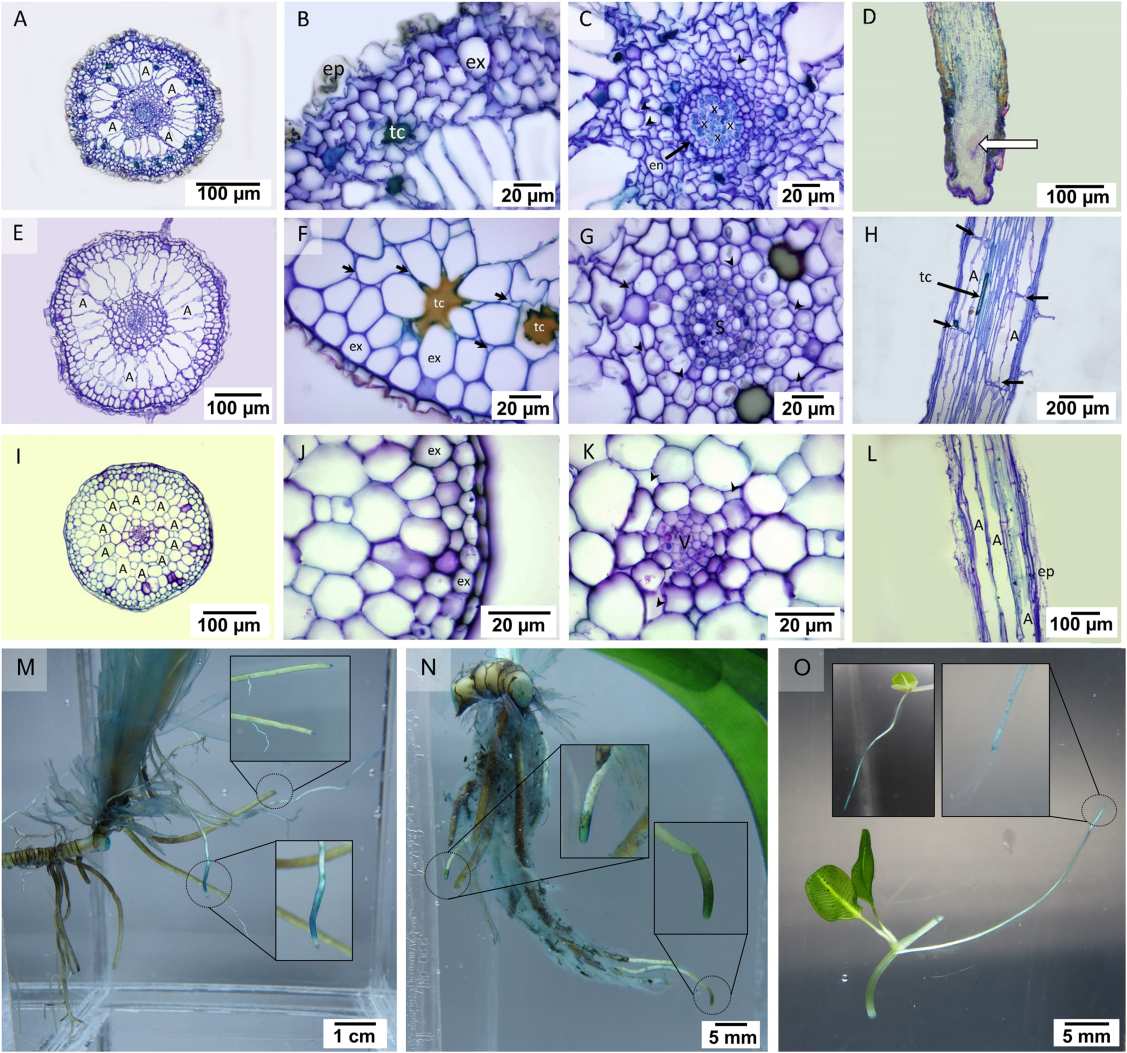
Root anatomical characteristics and localization of oxygen loss. Root anatomical characteristics of seagrasses. (A–D) *Cymodocea rotundata*, (E–H) *Thalassia hemprichii* and (I–L) *Halophila ovalis* and their localization of oxygen loss (M–O): (A) Transverse section of *C. rotundata* root. (B) The exodermis layers in the outer cortex. (C) The inner cortex with intercellular space (arrow head). (D) Root tip (E) Transverse section of *T. hemprichii* root. (F) The outer cortex with intercellular space (arrow). (G) Stele and the inner cortex with intercellular space (arrow head). (H) Lacunal diaphragm in the root (arrow). (I) Transverse section of *H. ovalis* root. (J) Exodermis layers in the outer cortex (K) Stele and inner cortex with intercellular space (arrow head) (L) Diaphragm-free air lacunae root. (M) *Cymodocea rotundata* root with ROL detected (blue staining) at the root tip. The younger root (lower inset) showed higher oxidation than the older root (upper inset). (N) *Thalassia hemprichii* root with ROL detected at the root tip. The left inset shows the younger root, and the right inset shows the older root. (O) Oxidized root tip of the second youngest ramet (right inset) and the apical ramet (left inset) of *Halophila ovalis*. Scale bars: 100 µm (A, D, E, I, L); 20 µm (B, C, F, G, J, K); 200 µm (H); 1 cm (M); 5 mm (N, O) A, Air lacuna; ep, epidermis; en, endodermis; ex, exodermis; s, stele; tc, tanniferous cell; x, xylem; v, vascular tissue.

In the transverse section of the root, small stele and distinguishable cortical layers were seen (Fig. 2). *Cymodocea rotundata* and *T. hemprichii* showed a similar structure of the thickened-wall exodermis in the outer cortex and radial arrangement of the narrowly ovoid-shaped air lacuna in the middle cortex (Figs. 2A, 2E), while in *H. ovalis*, the air lacuna was round in shape (Fig. 2I). In *C. rotundata*, the thin-walled epidermal layer was found covering the outer cortex. The main structure of the outer cortex consisted of 4–6 layers of compact parenchyma cells with the thickened wall of exodermis at the outermost layer (Fig. 2B). The large air lacunae in the middle cortex were adjacent to the loose parenchymal tissue and small stele of the inner cortex (Fig. 2C). The longitudinal section of the *C. rotundata* root tip showed compact parenchyma with a lack of air lacuna similar to the observation in *T. hemprichii* and *H. ovalis* root tip (Fig. 2D). The root structure of *T. hemprichii* showed the same tissue arrangement as in *C. rotundata* but displayed a larger cell size. The innermost area of the outer cortex consisted of loose parenchyma as observed in the inner cortex (Fig. 2F). The vascular tissue arrangement inside the stele of *T. hemprichii* root showed the same pattern as in *C. rotundata* (Fig. 2G). The longitudinal section demonstrated the tubular shape of air lacuna and the position of the lacunal diaphragm, which divided air lacunae into chambers (Fig. 2H). The roots of *H. ovalis* are unique among the three species as indistinct cortical regions with a prominent single row of round-shape air lacunae, and a spoke-like arrangement of parenchyma in the middle cortex was observed (Fig. 2I). A thin epidermis was found adjacent to the thick-walled exodermis (Fig. 2J). In addition, the smallest stele among three seagrass species was observed in *H. ovalis*. Their steles were surrounded by thin-walled parenchyma of the inner cortex (Fig. 2K). Non-diaphragm air lacunae type was found in *H. ovalis* (Fig. 2L).

The quantifiable parameters associated with root anatomy are displayed in Table 1 (see complete dataset in Supplemental Dataset and ANOVA results in Table S1). The root sizes of *C. rotundata* and *T. hemprichii* were twice as thick as that of *H. ovalis*. The highest epidermal layers thickness was found in *T. hemprichii* (DMRT test), whereas *C. rotundata* and *H. ovalis* displayed comparable values. The small size of the *H. ovalis* root resulted in significantly lower thickness in all cortical parameters compared to *C. rotundata* and *T. hemprichii* (DMRT test). Nevertheless, the outer: middle: inner cortex ratios were 3:2:5 in *C. rotundata*, 2:3:5 in *T. hemprichii* and 3:3:4 in *H. ovalis*, respectively. The highest stele diameter and air lacuna number were found in *T. hemprichii* (DMRT test). However, the cortex and stele thickness ratio showed no statistical difference among species. Tanniferous cells were present in *C. rotundata* and *T. hemprichii* but not in *H. ovalis*.

**Table 1:** Parameters associated with the anatomical features of leaves, rhizomes, and roots of three seagrass species.

Anatomical parameters		*C. rotundata*	*T. hemprichii*	*H. ovalis*
Root	Root thickness (µm)	701.97 ± 36.11^a^	690.86 ± 21.18^a^	333.93 ± 6.50^b^
	Epidermal layer thickness (µm)	6.34 ± 0.38^b^	18.18 ± 1.29^a^	10.79 ± 2.05^b^
	Total cortex thickness (µm)	333.67 ± 17.07^a^	298.78 ± 10.27^a^	146.45 ± 9.98^b^
	Outer cortex thickness (µm)	102.03 ± 25.51^a^	64.54 ± 3.75^ab^	43.28 ± 4.76^b^
	Middle cortex thickness (µm)	78.06 ± 4.57^a^	77.60 ± 2.57^a^	48.49 ± 4.18^b^
	Inner cortex thickness (µm)	153.58 ± 20.98^a^	156.64 ± 13.74^a^	54.69 ± 1.53^b^
	Ratio of outer: middle: inner cortex	3: 2: 5	2: 3: 5	3: 3: 4
	Stele diameter (µm)	42.99 ± 1.22^b^	50.85 ± 1.98^a^	23.86 ± 2.01^c^
	Ratio of cortex and stele thickness	0.13 ± 0.01^a^	0.17 ± 0.01^a^	0.17 ± 0.02^a^
	Air lacuna numbers	27.67 ± 1.20^b^	33.00 ± 1.00^a^	9.67 ± 0.67^c^
	Air lacuna shape	Irregular ovoid	Rectangular	Round
	Tannin cells	Present	Present	Absent
Rhizome	Rhizome thickness (µm)	1555.76 ± 283.05^a^	4941 ± 255.53^b^	1179.34 ± 124.88^a^
	Epidermal layer thickness (µm)	23.33 ± 3.21^ab^	29.20 ± 3.75^a^	17.48 ± 2.74^b^
	Total cortex thickness (µm)	690.762 ± 137.24^a^	792.62 ± 87.26^a^	526.62 ± 18.92^a^
	Outer cortex thickness (µm)	266.38 ± 48.83^a^	231.64 ± 7.09^a^	103.68 ± 10.94^b^
	Middle cortex thickness (µm)	336.46 ± 77.61^a^	428.11 ± 91.80^a^	330.07 ± 30.14^a^
	Inner cortex thickness (µm)	87.92 ± 17.91^a^	132.877 ± 8.61^a^	92.90 ± 3.88^ab^
	Ratio of outer: middle: inner cortex	4: 5: 1	3: 5: 2	2: 6: 2
	Stele diameter (µm)	147.43 ± 25.05^ab^	209.13 ± 23.16^a^	117.42 ± 12.66^c^
	Ratio of cortex and stele thickness	0.22 ± 0.03^a^	0.26 ± 0.01^a^	0.26 ± 0.02^a^
	Rhizome porosity (%)	32.16 ± 5.14^a^	30.44 ± 5.65^a^	44.61 ± 4.86^a^
	Air lacuna numbers	100.00 ± 10.39^a^	125.33 ± 11.57^a^	18.00 ± 0.00^b^
	Air lacuna shape	Narrowly ovoid	Narrowly ovoid	Round
	Tannin cells	Present	Present	Absent
Leaf	Leaf thickness (µm)	170.65 ± 11.62^a^	172.32 ± 1.35^a^	35.38 ± 2.14^b^
	Abaxial epidermal cell thickness (µm)	15.66 ± 1.62^ab^	13.23 ± 0.48^b^	17.49 ± 0.74^a^
	Adaxial epidermal cell thickness (µm)	14.39 ± 0.50^a^	9.25 ± 1.90^b^	17.89 ± 1.42^a^
	Mesophyll thickness (µm)	142.22 ± 14.44^a^	112.27 ± 1.27^b^	0.00 ± 0.00^c^
	Number of cell layer in mesophyll	6.00 ± 0.00^a^	5.67 ± 0.33^a^	0.00 ± 0.00^b^
	Vascular bundle numbers	12.67 ± 0.33^a^	9.67 ± 0.33^b^	1.00 ± 0.00^c^
	Air lacuna numbers	68.67 ± 3.28^a^	35.67 ± 1.20^b^	4.67 ± 3.33^c^
	Leaf porosity (%)	64.27 ± 3.67^a^	61.97 ± 1.78^a^	12.77 ± 1.16^b^
	Position of air lacuna	Mesophyll	Mesophyll	Midvein
	Tannin cells	Present	Present	Absent

**Note:**

Data above shows mean ± standard error (S.E.). Comparison of the mean values was analyzed using Duncan’s Multiple Range Test (DMRT). Values for each measurement with the same letter are not significantly different at *p* = 0.05.

Seagrass roots exhibited a difference in porosity among root zones and species (Table 2, see Fig. 1A for the indications of root zones). Significant effects of species (two-way ANOVA, df = 2, MS = 162.17, F = 20.72, *p* = 0.000001), root zone (two-way ANOVA, df = 2, MS = 1247.43, F = 159.37, p = 0.000000) and interactions between the two factors (two-way ANOVA, df = 4, MS = 105.47, F = 13.47, *p* = 0.000001) were detected in root porosity whereas significant effects of species (two-way ANOVA, df = 2, MS = 162450.23, F = 20.85, *p* = 0.000001) was detected in root diameter. Well-developed air lacunae were present in the middle and basal zones in all three seagrass species, while the root apical zone consisted of compact parenchyma with minimal intercellular space. Porosity showed an increasing trend towards the basal part. The highest root porosity was detected in the basal zone of *T. hemprichii* root (up to 27.76%), whereas the lowest root porosity was detected in the apical zone of *H. ovalis* (<1%). The root porosity in *H. ovalis* increased with greater root size. Still, this trend was not observed in *C. rotundata* and *T. hemprichii* as there was no significant difference in root diameter among the three zones in these two seagrasses. However, root diameter in *H. ovalis* exhibited the highest diameter at the basal zone and significantly decreased towards the apical zones.

**Table 2:** Root porosity and root diameter at the apical, middle and basal parts of three seagrass species.

Root zones	*Cymodocea rotundata*		*Thalassia hemprichii*		*Halophila ovalis*	
	Porosity (%)	Root diameter (µm)	Porosity (%)	Root diameter (µm)	Porosity (%)	Root diameter (µm)
Apical	5.05 ± 1.06^b^	328.13 ± 14.46^a^	1.55 ± 0.18^c^	514.82 ± 60.75^a^	0.95 ± 0.50^c^	216.43 ± 10.85^c^
Middle	12.25 ± 1.60^a^	387.81 ± 61.20^a^	20.37 ± 1.17^b^	498.09 ± 62.63^a^	16.19 ± 0.69^b^	304.46 ± 20.92^b^
Basal	13.35 ± 1.85^a^	440.73 ± 20.96^a^	27.76 ± 1.96^a^	506.31 ± 25.93^a^	18.89 ± 0.56^a^	381.69 ± 25.09^a^

**Note:**

Data above shows mean ± standard error (S.E.). A comparison of the mean values measured in different root zones within the same species was analyzed using Duncan’s Multiple Range Test (DMRT). Values for each measurement with the same letter are not significantly different at *p* = 0.05.

Detection of oxygen leakage by methylene blue oxidation showed blue-colour staining at the tip of the mature root of *C. rotundata*, *T. hemprichii* and *H. ovalis* (Figs. 2M–2O). The younger root tissues of all three seagrass species appear to be an essential site of oxygen leakage where higher oxidization of methylene blue, visualized as darker and larger stained area, was detected (Figs. 2M–2O).

#### Rhizome anatomy

**Figure 3:**
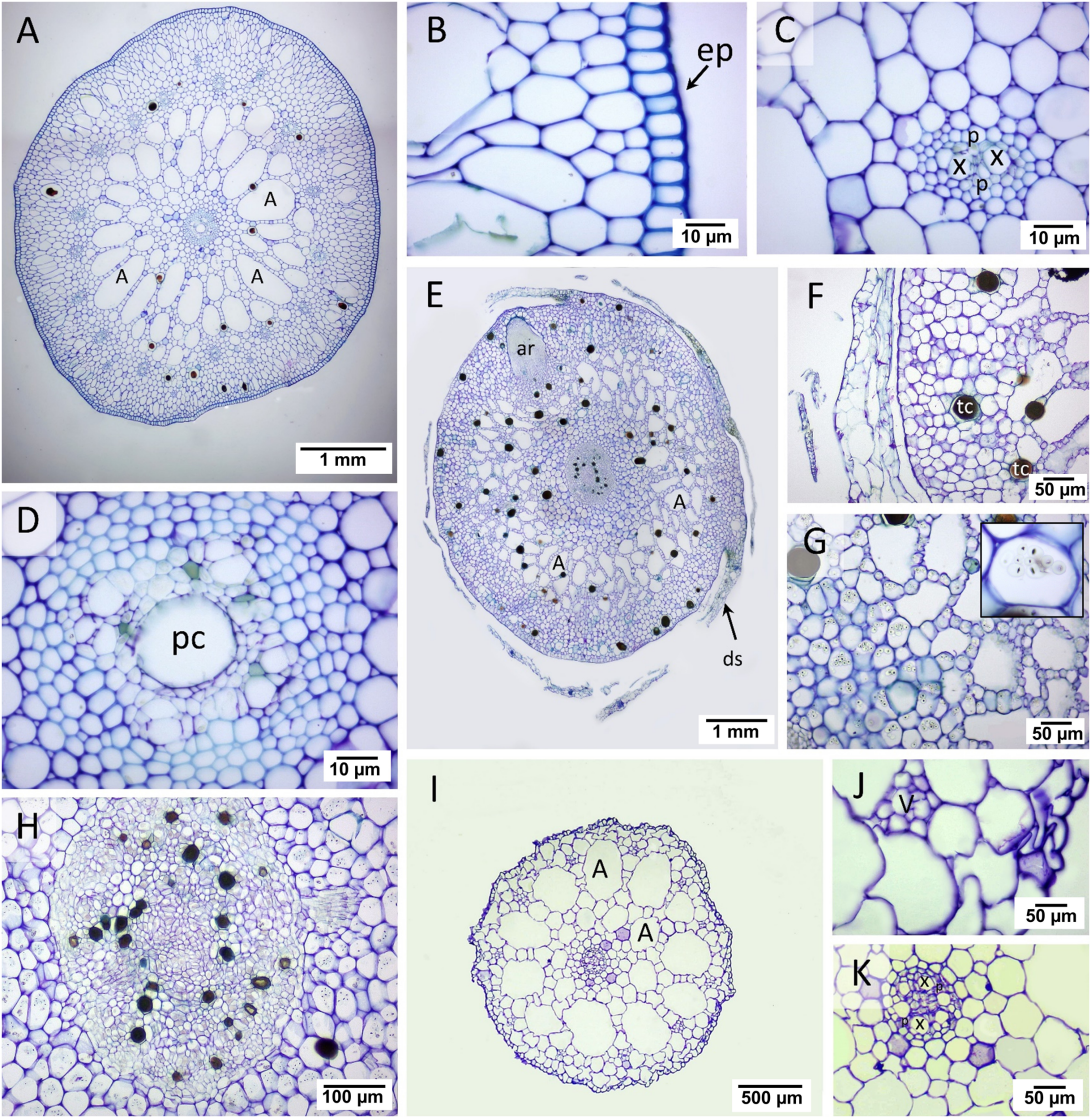
Rhizome anatomical characteristics. (A–D) *Cymodocea rotundata*, (E–H) *Thalassia hemprichii* and (I–K) *Halophila ovalis*. (A) Transverse section of *Cymodocea rotundata* rhizome. (B) Epidermis and the outer cortex. (C) The inner part of the outer cortex. (D) The stele with pith cavity. (E) Transverse section of *Thalassia hemprichii* rhizome. (F) The outer cortex. (G) The connecting zone of the middle and inner cortex with starch-accumulating cells (inset). (H) Stele (I) Transverse section of *H. ovalis*. (J) The outer cortex (K) Stele. Scale bars: 1 mm (A, E); 10 µm (B, C, D); 50 µm (F, G, J, K); 100 µm (H); 500 µm (I) A, Air lacuna; ar, adventitious root; ds, decayed leaf sheath; ep, epidermis; p, phloem; pc, pith cavity; tc, tanniferous cell; x, xylem.

Two regions consisting of stele and cortex were observed in the transverse rhizome sections of all three seagrasses. The cortex displayed three distinct regions, outer, middle, and inner cortex (Figs. 3A, 3E, 3I). In *C. rotundata*, the exodermis consisted of 2–3 layers of the compact parenchyma with thicken-wall cells, covered by the thin cuticle epidermis (Fig. 3B). The outer cortex of *C. rotundata* was composed of thin-walled parenchyma cells with sizeable intercellular space and schizogenous cavities arranged concentrically (Figs. 3B, 3C). High porosity was seen in the middle cortex as numerous large air lacunae and aerenchyma. At the same time, the inner cortex was composed of thin-walled compact parenchyma surrounding small steles with the poorly-lignified vessel and sieve elements in vascular tissue (Figs. 3A, 3D). Pith cavity was sometimes found in the old rhizome. The rhizome of *T. hemprichii* was usually found covered with decayed leaf sheaths (Fig. 3E). The thick cuticle epidermis covered several layers of thin-walled parenchyma in the outer cortex (Fig. 3F). The middle cortex was characterized by high porosity and occupied many air lacunae, aerenchyma and starch storage parenchyma. The inner cortex consisted of compact thin-walled starch storage parenchyma (Fig. 3G). *Thalassia hemprichii* had the largest stele among the three species with dense thin-walled vascular tissue (Fig. 3H). *Halophila ovalis* rhizome was mainly composed of thin epidermis, compact parenchyma and large air lacunae (Fig. 3I). The outer cortex consisted of thin-walled parenchyma and peripheral vascular bundles (Fig. 3J). The middle cortex consisted of two rows of well-developed air lacunae (the outer row is larger than the inner row). The inner cortex consisted of compact thin-walled parenchyma enclosing a small stele. The thickened wall endodermis was adjacent to the stele with a concentric xylem vessel arrangement (Fig. 3K).

The quantifiable parameters associated with rhizome anatomy are displayed in Table 1 (see complete dataset in Supplemental Dataset and ANOVA results in Table S1). The largest rhizome diameter and epidermal layer thickness were found in *T. hemprichii*, followed by *C. rotundata* and *H. ovalis*, respectively (DMRT test). *Halophila ovalis* had the thinnest outer cortex compared to *C. rotundata* and *T. hemprichii* (DMRT test). The outer: middle: inner cortex thickness ratios were 4:5:1 in *C. rotundata*, 3:5:2 in *T. hemprichii* and 2:6:2 in *H. ovalis*. The stele diameters of *C. rotundata* and *T. hemprichii* were not significantly different, while the smallest stele diameter was found in *H. ovalis* (DMRT test). The ratios of cortex: stele thickness of three seagrass species were not statistically different. While *C. rotundata* and *T. hemprichii* showed air lacuna numbers higher than a hundred, *H. ovalis* had only 18 air lacunae on average. Still, the percentage of porosity did not differ among species.

#### Leaf anatomy

**Figure 4:**
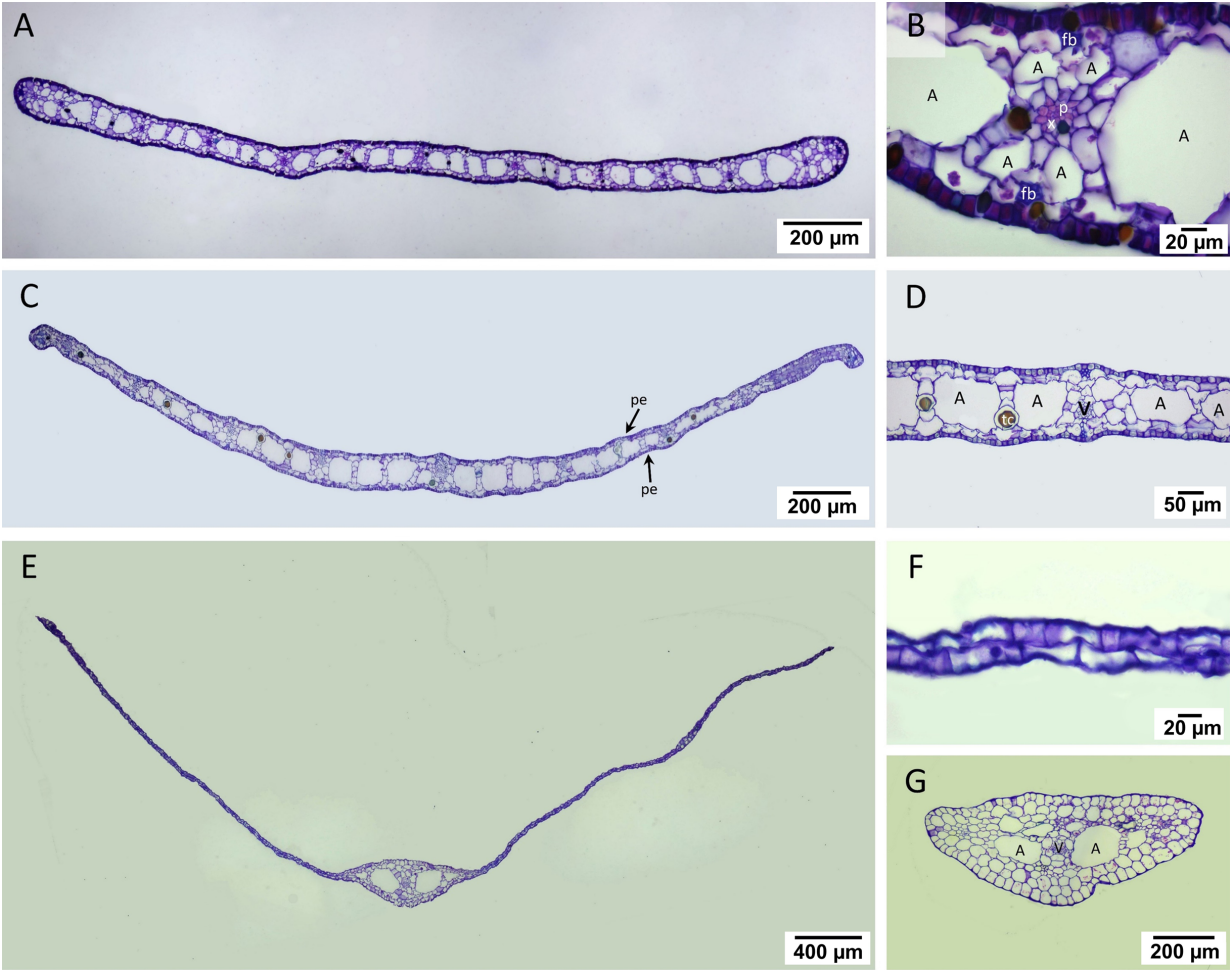
Leaf anatomical characteristics. **Leaf anatomical characteristics.** (A–B) *Cymodocea rotundata*, (C–D) *Thalassia hemprichii* and (E–G) *Halophila ovalis*. (A) Transverse section of *Cymodocea rotundata* lamina. (B) Structure of mesophylls. (C) Transverse section of *Thalassia hemprichii* lamina. (D) Structure of mesophylls. (E) Transverse section of *Halophila ovalis* lamina. (F) Lamina of *H. ovalis* (G) Petiole of *H. ovalis*. Scale bars: 200 µm (A, C, G); 20 µm (B, F); 50 µm (D); 400 µm (E) A, Air lacuna; pe, photosynthetic epidermis; fb, fiber strands; p, phloem; tc, tanniferous cell; v, vascular tissue; x, xylem.

The laminar of *T. hemprichii* and *C. rotundata* showed well-developed mesophyll, which was more than four times thicker than that of *H. ovalis* (Figs. 4A, 4C, 4E, Table 1). In *T. hemprichii* and *C. rotundata*, the mesophyll layer was primarily occupied by several air lacunae, separated into chambers by parenchyma cells. Meanwhile, the thin laminar of *H. ovalis* consisted of two cell layers with a few central air lacunae in the midvein. The epidermis of three seagrasses species is photosynthetically-active as indicated by the bulk of chloroplasts inside (Figs. 4B, 4D, 4F). *Cymodocea rotundata* and *H. ovalis* showed similar epidermal cell thickness on both sides. In contrast, the adaxial epidermal cell of *T. hemprichii* was thin (approx. 9.25 µm) compared to the abaxial surface (13.23 µm) (Table 1). The midvein and petiole of *H. ovalis* exhibited similar cell arrangements. The vascular bundle was located at the center with a large air lacuna flanking at both sides (Fig. 4G).

Comparative leaf anatomical features (Table 1, see complete dataset in Supplemental Dataset and ANOVA results in the Table S1) revealed the highest mesophyll thickness in *C. rotundata*, with the highest vascular bundle and air lacuna number (DMRT test). While the percentage of leaf porosity of *C. rotundata* (64.27%) and *T. hemprichii* (61.97%) did not differ from each other, they were much higher than the value observed in *H. ovalis* (DMRT test). *Halophila ovalis* laminar has a single central vascular bundle with low porosity, lacking air lacuna and mesophyll.

### Comparing seagrass gas exchange and electron transport rates in contrasting oxygen levels in the belowground compartment

**Figure 5:**
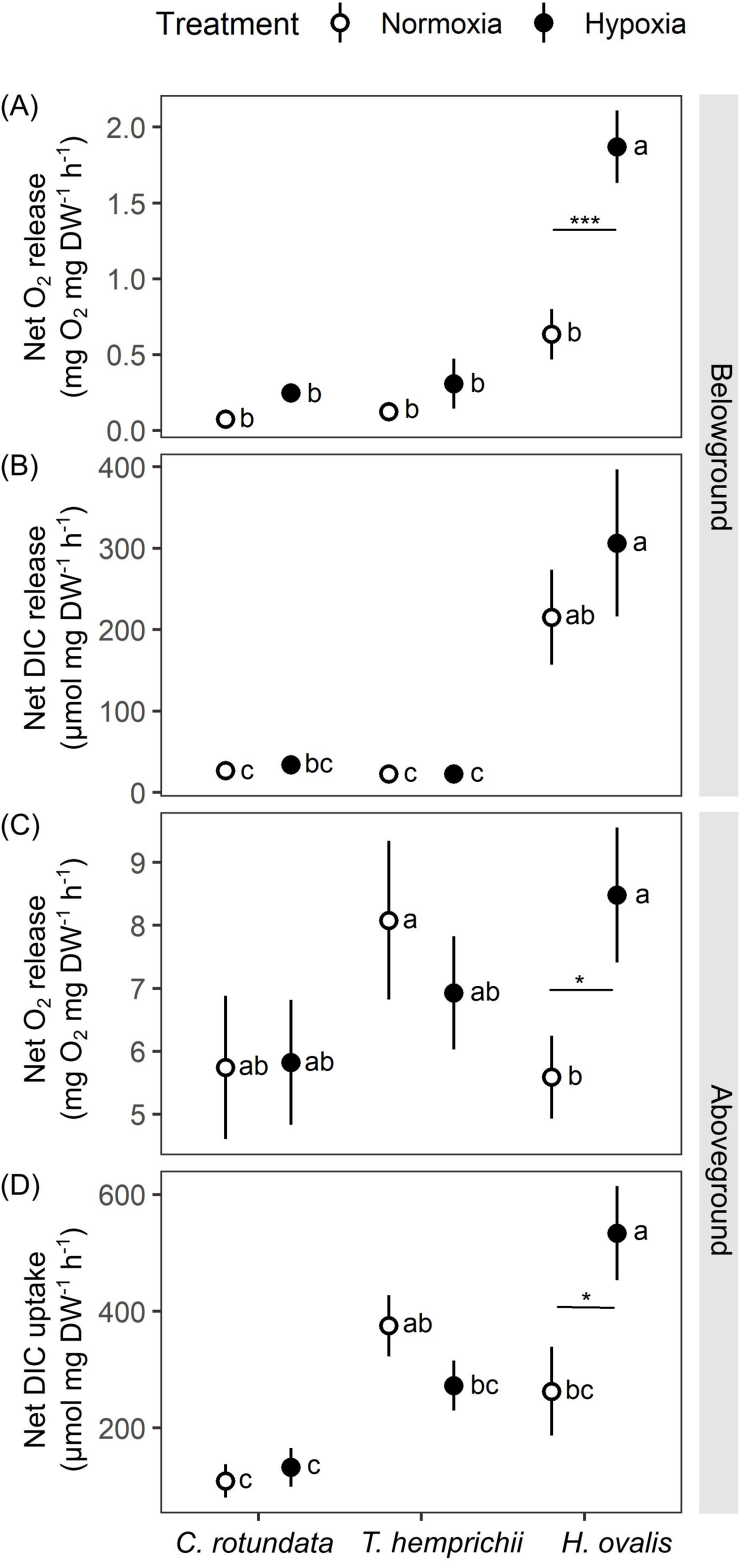
Net oxygen and dissolved inorganic carbon (DIC) exchange rates in belowground (BG) and aboveground (AG) tissues in three tropical seagrasses exposed to contrasting oxygen levels in the belowground compartment. (A) Net oxygen release rates in BG tissues. (B) Net DIC release rates in BG tissues. (C) Net oxygen release rates in AG tissues. (D) Net DIC uptake rates in AG tissues. Data shows mean ± standard error (S.E.). A comparison of the mean values was analyzed using Tukey’s HSD test. Values for each measurement with the same letter are not significantly different at *p* = 0.05. The difference between normoxia and root hypoxia is indicated by asterisks (*p ≤ 0.05, ***p ≤ 0.001).

Both oxygen and dissolved inorganic carbon (DIC) exchange rates showed variations depending on species and initial dissolved oxygen (DO) level in the belowground (BG) compartment (Fig. 5). Plant-free setup (the negative control) displayed minimal changes in DO and pH (Table S2 and see complete dataset in Supplemental Dataset), suggesting that the biological processes associated with the plant materials were the main contributor to the modification of the seawater chemistry observed in this study.

Significant effects of species (repeated ANOVA, df = 2, MS = 9.06, F = 27.36, *p* = 0.000000), DO level (repeated ANOVA, df = 1, MS = 5.06, F = 35.37, *p* = 0.000000) and interactions of the two factors (repeated ANOVA, df = 2, MS = 2.24, F = 15.67, p = *0*.000001) on net oxygen release rates from BG tissue were detected. The net oxygen release rates from BG tissue displayed no difference among species when the BG tissues were incubated in normoxia condition (Fig. 5A). Hypoxia condition significantly enhanced the net oxygen release rate by almost three times in BG tissues of *Halophila ovalis* (Tukey’s HSD test). A marginal increasing trend was also observed in other seagrass species, but it was not statistically significant. Consequently, the highest net oxygen release rate from BG tissue was found in *H. ovalis* in hypoxia conditions (Fig. 5A). Changes in DO in the BG compartment of the plant-free setup were 0.00 ± 0.00 mg L^−1^ in normoxia and 0.01 ± 0.04 mg L^−1^ in hypoxia treatment, corresponding to an oxygen release rate of 0.00 ± 0.00 mg h^−1^ in both treatments.

Net DIC release rates in the BG tissue, representing estimates of net respiratory rates, were found to be species-specific (Fig. 5B, repeated ANOVA, df = 2, MS = 439,374.6, F = 13.67, *p* = 0.000048). *H. ovalis* displayed the highest rates of net DIC release (Tukey’s HSD test), whereas the values measured in the other two seagrass species were comparable. Lowering DO levels did not affect net DIC release in any seagrass species (Fig. 5B). The pH changes in the BG compartment of the plant-free setup were 0.03 ± 0.01 in normoxia and 0.04 ± 0.01 in hypoxia treatment, corresponding to DIC release rate of 0.94 ± 0.23 and 1.56 ± 0.55 µmol h^−1^ in normoxia and hypoxia treatments, respectively.

While repeated ANOVA did not show the significant effects of species and DO level (Fig. 5C), Tukey’s HSD test showed that root hypoxia enhanced the net oxygen release in the aboveground (AG) tissues in *H. ovalis* but not in other seagrasses. Changes in DO in the AG compartment of the plant-free setup were 0.04 ± 0.02 mg L^−1^ in normoxia and 0.05 ± 0.03 mg L^−1^ in root hypoxia treatment, corresponding to the oxygen release rate of 0.04 ± 0.01 mg h^−1^ and 0.04 ± 0.03 mg h^−1^ in normoxia and hypoxia treatments, respectively.

Significant effects of species (repeated ANOVA, df = 2, MS = 494,762, F = 9.99, *p* = 0.000406) and interactions of species and DO level (repeated ANOVA, df = 2, MS = 216,388, F = 8.4837, *p* = 0.001065) on net DIC uptake rates in the AG tissue were detected. Net DIC uptake rates representing estimates of net photosynthetic carbon uptake were species-specific (Fig. 5D). As a general trend, the lowest DIC consumption was found in *C. rotundata* (Tukey’s HSD test), whereas the values measured in *H. ovalis* and *T. hemprichii* were similar. Lowering DO in the BG compartment had a stimulating effect on the net DIC uptake rate in AG tissues of *H. ovalis* (Tukey’s HSD test). Still, no significant effect was observed in the other two seagrasses. The pH changes in the AG compartment in the plant-free setup were 0.01 ± 0.01 in normoxia and 0.01 ± 0.01 in hypoxia treatment, corresponding to DIC release rate of −4.92 ± 0.01 and −4.74 ± 3.97 µmol h^−1^ in normoxia and hypoxia treatments, respectively.

The electron transport rates through photosystem II (ETR) were affected by DO in the BG compartment in a species-specific manner as significant effects of species (repeated ANOVA, df = 2, MS = 6,645.2, F = 34.18, *p* = 0.000003) and interactions between species and DO (repeated ANOVA, df = 2, MS = 1,655.9, F = 6.986, *p* = 0.007174) were detected. While root hypoxia did not induce any significant change in *C. rotundata* and *H. ovalis*, it was found to inhibit the ETR of *T. hemprichii* by approximately 30% (Table 3, Tukey’s HSD test).

**Table 3:** Photosystem II electron transport rates after incubation in different oxygen and temperature levels.

Species	Treatments	PSII electron transport rates (µmol m^−2^s^−1^)
*Cymodocea rotundata*	Normoxia	93.33 ± 5.97^a^
	Hypoxia	107.25 ± 3.47^a^
*Thalassia hemprichii*	Normoxia	92.41 ± 5.09^a^
	Hypoxia	63.48 ± 5.60^b^
*Halophila ovalis*	Normoxia	48.64 ± 4.27^b^
	Hypoxia	57.84 ± 9.63^b^
*Cymodocea rotundata*	30 °C	89.98 ± 4.57^a^
	40 °C	61.93 ± 2.54^b^
*Thalassia hemprichii*	30 °C	105.78 ± 16.36^a^
	40 °C	56.81 ± 4.75^b^
*Halophila ovalis*	30 °C	56.64 ± 3.39^b^
	40 °C	36.69 ± 5.14^c^

**Note:**

The data shown above represent the mean ± standard error (S.E.). A comparison of the mean values was analyzed using Tukey’s HSD test. Values for each measurement with the same letter are not significantly different at *p* = 0.05.

### Comparing seagrass gas exchange and electron transport rates at optimal and high temperatures

**Figure 6:**
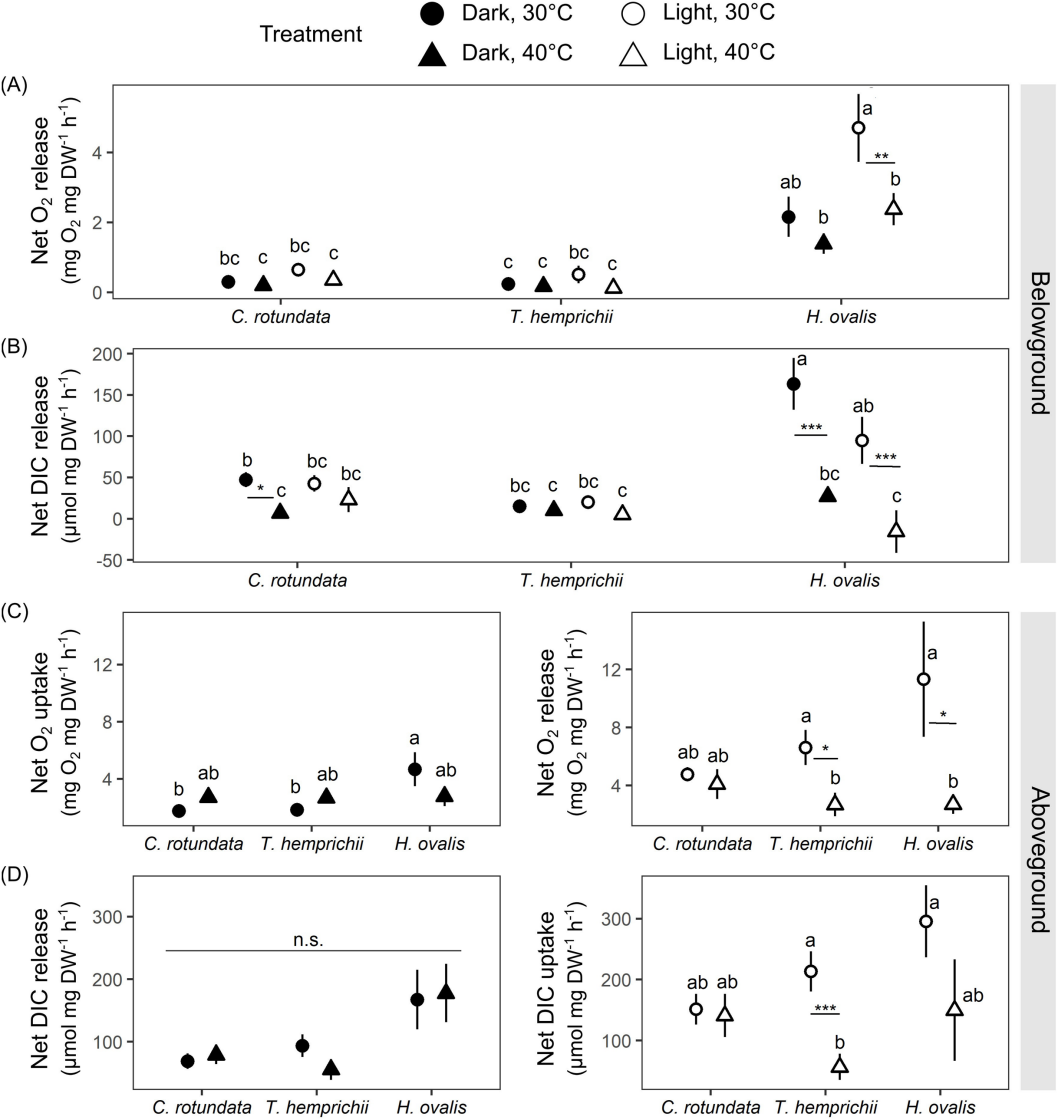
Net oxygen and dissolved inorganic carbon (DIC) exchange rates in belowground (BG) and aboveground (AG) tissues in three tropical seagrasses in darkness and in the light at different temperatures (30 °C and 40 °C). (A) Net oxygen release rates in BG tissues. (B) Net DIC release rates in BG tissues. (C) Net oxygen exchange rates in AG tissues, the values measured in darkness represent net O_2_ uptake rates (left), and the values measured in the light represent net O_2_ release rates (right). (D) Net DIC exchange rates in AG tissues, the values measured in darkness represent net DIC release rates (left), and the values measured in the light represent net DIC uptake rates (right). Data shows mean ± standard error (S.E.). A comparison of the mean values was analyzed using Tukey’s HSD test. Values for each measurement with the same letter are not significantly different at *p* = 0.05. n.s., not statistically significant by analysis of variance (ANOVA) and p Tukey’s HSD test. The difference between 30 °C and 40 °C is indicated by asterisks (*p ≤ 0.05, **p ≤ 0.01, *** *p*≤ 0.001).

Both oxygen and DIC exchange rates showed variations depending on species and temperatures (Fig. 6). Similar to the observation in the previous experiment, plant-free setup (the negative control) displayed minimal changes in DO and pH (Table S2, see complete dataset in Supplemental Dataset).

There were significant effects of species (repeated ANOVA, df = 2, MS = 72.57, F = 34.99, *p* = 0.000000), temperature (repeated ANOVA, df = 1, MS = 12.55, F = 6.05, *p* = 0.017177), light (repeated ANOVA, df = 1, MS = 14.73, F = 19.87, *p* = 0.000043), interactions between light and species (repeated ANOVA, df = 2, MS = 8.33, F = 11.23, *p* = 0.000086) and interactions between light and temperature (repeated ANOVA, df = 1, MS = 3.50, F = 4.72, *p* = 0.034275) on net oxygen release rates from BG tissues(Fig. 6A). Similar to the response observed in the previous experiment, *H. ovalis* had the highest net oxygen release rates from BG tissues (Tukey’s HSD test). In contrast, those in *C. rotundata* and *T. hemprichii* were comparable (Fig. 6A). Net oxygen release rates in BG tissues of *C. rotundata* and *T. hemprichii* were not affected by temperature and did not differ between those measured in darkness or in the light. On the contrary, a significant inhibitory effect of warming was detected in *H. ovalis* in the light (Tukey’s HSD test). In darkness, changes in DO in the BG compartment of the plant-free setup were 0.36 ± 0.13 mg L^−1^ in 30 °C and 0.15 ± 0.05 mg L^−1^ in 40 °C treatments, corresponding to an oxygen release rates of 0.02 ± 0.01 mg h^−1^ and 0.01 ± 0.00 mg h^−1^ at 30 °C and 40 °C, respectively. In the light, changes in DO in the BG compartment of the plant-free setup were 0.20 + 0.09 mg L^−1^ in 30 °C and 0.41 ± 0.16 mg L^−1^ in 40 °C treatments, corresponding to oxygen release rates of 0.01 ± 0.01 mg h^−1^ and 0.03 ± 0.01 mg h^−1^ at 30 °C and 40 °C, respectively (Table S2, see complete dataset in Supplemental Dataset).

There were significant effects of species (repeated ANOVA, df = 2, MS = 28,313.3, F = 8.67, *p* = 0.000587), temperature (repeated ANOVA, df = 1, MS = 81,855.4, F = 25.06, *p* = 0.000007), light (repeated ANOVA, df = 1, MS = 7,275.4, F = 4.13, *p* = 0.047545), interactions between temperature and species (repeated ANOVA, df = 2, MS = 34,536.7, F = 10.57, *p* = 0.000148) and interactions between light and species (repeated ANOVA, df = 2, MS = 11,050.4, F = 6.27, *p* = 0.003724) on the net DIC release rates in BG tissue (Fig. 6B). The net DIC release rates in BG tissue, representing estimates of net respiratory rates, were negatively affected by temperature (Fig. 6B). Net DIC release rates in darkness and in the light of *T. hemprichii* did not differ from each other and were not significantly affected by temperature. Net DIC release rates in the dark of *C. rotundata* was lower in 40 °C (Tukey’s HSD test), and both DIC release rates in darkness and in the light of *H. ovalis* were significantly inhibited by warming (Tukey’s HSD test). In darkness, changes in pH in the BG compartment of the plant-free setup were 0.01 ± 0.01 in 30 °C and 0.01 ± 0.01 in 40 °C treatments, corresponding to DIC release rate of 0.44 ± 0.22 µmol h^−1^ and 0.23 ± 0.74 µmol h^−1^ at 30 °C and 40 °C, respectively. In the light, changes in pH in the BG compartment of the plant-free setup were 0.01 + 0.01 in 30 °C and 0.02 ± 0.02 in 40 °C treatments, corresponding to DIC release rates of 0.55 ± 0.36 µmol h^−1^ and 0.97 ± 1.21 µmol h^−1^ at 30 °C and 40 °C, respectively (Table S2, see complete dataset in Supplemental Dataset).

Net oxygen exchange rates in AG tissues of all seagrass species exhibited different responses depending on species, light, and temperature treatments (Fig. 6C). Significant effects of species (two-way ANOVA, df = 2, MS = 14.49, F = 3.62, *p* = 0.033485) and interactions between species and temperature were detected (two-way ANOVA, df = 2, MS = 13.03, F = 3.26, *p* = 0.046300) in net oxygen uptake rates in AG tissues in darkness. Nevertheless, the temperature did not affect oxygen uptake rates in AG tissues when comparing the same species. In the light, significant effects of temperature were detected in the net oxygen release rates (two-way ANOVA, df = 1, MS = 291.39, F = 8.88, *p* = 0.004321). When comparing within the same species, net oxygen release rates of *T. hemprichii* and *H. ovalis* were significantly lower at 40 °C (Tukey’s HSD test), with the more adverse effect observed in *H. ovalis*. In darkness, changes in DO in the AG compartment of the plant-free setup were 0.07 ± 0.02 mg L^−1^ in 30 °C and 0.10 ± 0.005 mg L^−1^ in 40 °C treatments, corresponding to an oxygen uptake rates of 0.06 ± 0.02 mg h^−1^ and 0.08 ± 0.004 mg h^−1^ at 30 °C and 40 °C, respectively. In the light, changes in DO in the AG compartment of the plant-free setup were 0.03 ± 0.03 mg L^−1^ in 30 °C and 0.06 ± 0.05 mg L^−1^ in 40 °C treatments, corresponding to oxygen release rates of 0.02 ± 0.03 mg h^−1^ and 0.05 ± 0.04 mg h^−1^ at 30 °C and 40 °C, respectively (Table S2, see complete dataset in Supplemental Dataset).

The net DIC exchange rates in AG tissues showed a similar response pattern as observed in net oxygen exchange rates (Fig. 6D). Net DIC release rates in darkness differed depending on species (two-way ANOVA, df = 2, MS = 46,172.4, F = 5.75, *p* = 0.005643), whereas net DIC uptake rates in the light were affected by temperature (two-way ANOVA, df = 1, MS = 169,473, F = 7.82, *p* = 0.007268). DIC uptake in the light in *T. hemprichii* was significantly inhibited by warming (Tukey’s HSD test), whereas a decreasing trend was detected in *H. ovalis*. In darkness, changes in pH in the AG compartment of the plant-free setup were 0.00 ± 0.00 in 30 °C and 0.01 ± 0.00 in 40 °C treatments, corresponding to DIC release rate of 3.25 + 2.13 µmol h^−1^ and 3.66 ± 3.86 µmol h^−1^ at 30 °C and 40 °C, respectively. In the light, changes in pH in the AG compartment of the plant-free setup were 0.01 ± 0.00 in 30 °C and 0.01 ± 0.01 in 40 °C treatments, corresponding to DIC uptake rates of −6.33 ± 2.0 µmol h^−1^ and −5.75 ± 5.60 µmol h^−1^ at 30 °C and 40 °C, respectively (see complete dataset in Supplemental Dataset and ANOVA results in the Table S1).

There were significant effects of species (two-way ANOVA, df = 2, MS = 8,340.5, F = 37.84, *p* = 0.000000), temperature (two-way ANOVA, df = 1, MS = 18,801.2, F = 85.30, *p* = 0.000000) and interactions between the two factors (two-way ANOVA, df = 2, MS = 1,345.7, F = 6.105, *p* = 0.003692) on ETR. The ETRs measured at 40 °C were significantly lower than those measured at 30 °C in all species (Tukey’s HSD, Table 3).

## Discussion

In general, *Halophila ovalis* had greater metabolic activity compared to *Cymodocea rotundata*, and *Thalassia hemprichii*, manifested as higher gas exchange rates per unit weight in both the belowground (BG) and aboveground (AG) tissues. *Halophila* are colonizing species, characterized by relatively high growth and turnover rates (Kilminster et al., 2015; O’Brien et al., 2018; Lamit & Tanaka, 2021), while *Cymodocea* and *Thalassia* are considered opportunistic and persistent seagrasses, respectively (Kilminster et al., 2015; O’Brien et al., 2018). Previous observations reported similar aboveground productivity between *C. rotundata* and *T. hemprichii* (Cebrián et al., 1998; Yamamuro & Chirapart, 2005; Collier & Waycott, 2014) and higher aboveground productivity in *H. ovalis* (Yamamuro & Chirapart, 2005; Yucharoen et al., 2021). In the present study, *H. ovalis* also displayed unique responses among the three tested species seen as significant modulation of gas exchange under changing conditions. In contrast, the other two seagrasses showed primarily comparable responses. Such differences may be explained by life traits, ecophysiology, morphological and anatomical features (Kiswara et al., 2009; Kilminster et al., 2015; Buapet, Makkliang & Thammakhet-Buranachai, 2017; Phandee & Buapet, 2018; Martin et al., 2019). Examination of anatomical structures of the three seagrasses also highlights the uniqueness of *H. ovalis*, which may underpin variations in gas exchange responses under root hypoxia and warming.

### Species-specific anatomical features associated with gas exchange

The three seagrass species share certain anatomical features, mainly in the belowground tissues. Three regions of the cortex, the outer, middle and inner layers, were seen in roots and rhizomes of the three seagrass species, though less distinct in *H. ovalis*. These observations are consistent with previous studies (Roberts, McComb & Kuo, 1985; Connell, Colmer & Walker, 1999; Kuo & den Hartog, 2006; Purnama et al., 2018). The apparent well-developed air lacunae were found in the middle cortex. In rhizomes of all species, the middle cortex also occupies the largest space among the three cortical layers. The presence of air lacunae implies that the middle cortex of seagrass rhizome may act as belowground oxygen storage (Pedersen et al., 1998; Borum et al., 2005; Sand-Jensen et al., 2005). Similar observations were reported in the seagrasses *C. rotundata* and *H. ovalis* (Roberts, McComb & Kuo, 1985; Pedersen et al., 1998; Connell, Colmer & Walker, 1999; Kuo & den Hartog, 2006) and in other wetland plants (Cheng et al., 2020a; Armstrong et al., 2000; Soukup, Votrubová & Čížková, 2002; Matthews & Seymour, 2014). It has been suggested that tissue porosity is associated with tolerance to hypoxic conditions (Visser et al., 2000; Hasler-Sheetal et al., 2015; Cheng et al., 2020a). While the porosity of rhizome did not differ among species, the highest root porosity was found in *T. hemprichii*, followed by *H. ovalis* and *C. rotundata*. Additionally, the root porosity was found to vary along the root length with a decreasing trend towards root tips in all species, in line with previous reports (Colmer, 2003; Cheng et al., 2020a). The porosity values obtained in the present study are within a range reported in the seagrass *H. ovalis* (Roberts, McComb & Kuo, 1985; Connell, Colmer & Walker, 1999) and *Zostera muelleri* (Stafford-Bell, Chariton & Robinson, 2015). Staining by methylene blue confirmed that root tip is an important site for radial oxygen loss (ROL), especially in *C. rotundata* and *T. hemprichii* (Connell, Colmer & Walker, 1999; Sand-Jensen et al., 2005; Frederiksen & Glud, 2006; Pi et al., 2009; Martin et al., 2019). The root tissues above root tips and rhizome of all species showed the thickened-wall exodermis in the outer cortex with a tightened lamella. However, a thinner cortex layer and exodermis were observed in *H. ovalis*. It has been suggested that these features act as a barrier to ROL in the roots of seagrasses and other wetland plants (Connell, Colmer & Walker, 1999; Visser et al., 2000; Hose et al., 2001; Kuo & den Hartog, 2006; Ejiri & Shiono, 2019; Martin et al., 2019; Cheng et al., 2020a). A barrier to ROL plays a role in limiting excess oxygen loss from the roots and maintaining suitable oxygen levels within the root tissues (Yamauchi et al., 2018; Martin et al., 2019; Pedersen et al., 2021) and may explain undetected oxygen leakage from the rhizomes and other portions of the roots by methylene blue oxidation in this study. However, studies have shown that aging significantly influences ROL, metabolic rates and acclimatory responses in seagrasses (Martin et al., 2019; Rasmusson et al., 2019; Roucco et al., 2020). The extrapolation of our results obtained from the youngest mature ramets to the whole meadow must be taken with caution.

The laminar of *C. rotundata* and *T. hemprichii* showed distinctive mesophylls with well-developed numerous air lacunae, whereas that of *H. ovalis* consists of two layers of photosynthetic epidermis without mesophyll and with a few air lacunae present in the midvein. The anatomical features observed in the present study agree with previous reports in the same seagrass species (Kuo & den Hartog, 2006; Kuo, Cambridge & Kirkman, 2018) but contradict the results by Purnama et al. (2018), which displayed mesophyll without air lacunae in *T. hemprichii*. Nevertheless, it seems that most of the anatomical features in *C. rotundata*, *T. hemprichii* and *H. ovalis* are relatively consistent across the populations examined (Roberts, McComb & Kuo, 1985; Pedersen et al., 1998; Connell, Colmer & Walker, 1999; Kuo & den Hartog, 2006; Kuo, Cambridge & Kirkman, 2018; Purnama et al., 2018).

### Root hypoxia altered gas exchange and photosynthesis in *Halophila ovalis*

Our results showed that *H. ovalis* was the only seagrass in which oxygen release in BG tissues was responsive to the dissolved oxygen (DO) in the surrounding water, displaying an increase of more than two-fold in root hypoxia condition. Seagrasses resist highly reduced and hypoxic conditions in the sediment by creating an oxic microenvironment around the roots (Brodersen et al., 2018a). Oxygen release from roots depends on (1) transport of oxygen from photosynthetic tissue (Connell, Colmer & Walker, 1999; Frederiksen & Glud, 2006), (2) difference in oxygen partial pressure between root cells and environment (Pedersen et al., 1998; Visser et al., 2000; Cheng et al., 2020a) and (3) barrier of radial oxygen loss (ROL) (Pi et al., 2009; Martin et al., 2019; Cheng et al., 2020a; Pedersen et al., 2021). An increase in oxygen release in root tissues in *H. ovalis* appeared to follow the rise in net oxygen evolution and carbon uptake detected in the aboveground tissues. This highlights the role of signaling between aboveground and belowground tissues (Li, Testerink & Zhang, 2021) and active regulation of root oxygen release by photosynthesis (Connell, Colmer & Walker, 1999; Frederiksen & Glud, 2006). Although the electron transport rates (ETR) through PSII did not differ significantly between the two conditions, an increasing trend in root hypoxia was also seen in *H. ovalis*. Longitudinal oxygen transport from photosynthetic tissue is regulated by photosynthetic activity and facilitated by the shoot-root lacunae network. The roots of *H. ovalis* have diaphragm-free air lacuna and a thin cortex layer and exodermis. These anatomical features may explain the highest tendency for oxygen leakage from the roots in this seagrass species. Sand-Jensen et al. (2005) suggested that the short distance between the aboveground compartment and root tip and diaphragm-free air lacunae readily facilitates oxygen supply to wetland plants’ roots. On the contrary, both *C. rotundata* and *T. hemprichii* have tightened-lamella of exodermis and the unique structure of lacunal diaphragms was also observed in *T. hemprichii*. Lacunal diaphragm has previously been reported in seagrasses (Sand-Jensen, Prahl & Stokholm, 1982; Roberts, McComb & Kuo, 1984) and is commonly found in many wetland species (Colmer, 2003). These features may contribute in better regulation of lacunal gas movement and limitation of ROL in *C. rotundata* and *T. hemprichii* (Colmer, 2003). Other characteristics such as root thickness and ratio of cortex-to-stele have been proposed to play a role in ROL regulation (Pedersen et al., 2021). It has been suggested that thicker roots are less susceptible to ROL than thinner ones (Pedersen et al., 2021), corroborating our results. As comparable cortex-to-stele ratios were observed, this parameter may not be a suitable indicator for ROL potential in the tested seagrass species.

While net oxygen evolution and DIC uptake in the aboveground compartment are more likely associated with the photophysiology of seagrasses (Buapet, Gullström & Björk, 2013; Rasmusson et al., 2017; Kong et al., 2019), some investigations have suggested an interrelation to the leaf anatomy (Hemminga & Duarte, 2000; Kuo & den Hartog, 2006; Sugiura et al., 2009; Kuo, Cambridge & Kirkman, 2018). The epidermis covered by a thin cuticle is a significant site of photosynthesis in seagrasses (Kuo & den Hartog, 2006). While species with porous cuticle or subcuticular cavities such as *Thalassia* and *Cymodocea* (Kuo, Cambridge & Kirkman, 2018) may increase their exchange of nutrients and DIC through the leaves (Hemminga & Duarte, 2000; Sugiura et al., 2009), very thin laminar of *H. ovalis* might compensate for the lack of this feature (Maberly & Gontero, 2018), resulting in similar gas exchange rates in the aboveground tissues among species in normoxia condition.

Although all seagrass samples were collected from the same site, variations in the anatomical features and gas exchange responses observed in this experiment suggest differing sensitivity to oxygen levels. More effective barrier and low ROL in *C. rotundata* and *T. hemprichii* ensure sufficient supply of oxygen to their metabolically-demanding root apical meristem in oxygen-deprived environment coupled with the potential need for oxic microenvironment to prevent sulfide toxicity (Brodersen et al., 2016, 2018b; Martin et al., 2019). *H. ovalis* may be more susceptible to oxygen leakage under hypoxia. However, upregulation of photosynthesis to support oxygen demand in the belowground tissues in this seagrass species is possible and should be further investigated. It needs to be emphasized that our results can only represent the gas exchange capacity of seagrasses as diffusion resistance is expected to be much higher in the rhizosphere, where circulation is limited (Terrados et al., 1999).

### *Halophila ovalis* is highly sensitive to thermal warming

Photosynthetically-derived oxygen is diffused to the water column and/or transported to belowground tissue where it is stored in the lacunae and/or released to the rhizosphere (Borum et al., 2006; Larkum et al., 2017). It has been established that oxygen release in BG tissue of seagrasses is associated with photosynthesis and is thus light-dependent (Pedersen et al., 1998; Connell, Colmer & Walker, 1999; Colmer, 2003; Greve, Borum & Pedersen, 2003; Frederiksen & Glud, 2006; Brodersen et al., 2016; Martin et al., 2019). This seems to be the case in BG tissues of *H. ovalis*, which showed lower oxygen release rates in darkness compared to the rates in the light whereas those of *C. rotundata* and *T. hemprichii* were not affected by light availability. These findings suggest *C. rotundata* and *T. hemprichii* may have greater overall capacity for oxygen storage. Pedersen et al. (1998) suggested that the storage capacity in the leaf lacunae determines the ability of the seagrass leaves to maintain their oxic microenvironment during nighttime whereas Greve, Borum & Pedersen (2003) suggested that the water column is a more important source of oxygen than photosynthesis at nighttime. Regardless of the oxygen source, higher porosity in the leaves of *C. rotundata* and *T. hemprichii* found in the present study may facilitate oxygen storage and subsequent transfer to the belowground tissues in darkness.

Oxygen release in the belowground tissue of *H. ovalis* decreased significantly at 40 °C, while other species remained unaffected. It is known that high temperature has a stimulating effect on cellular respiration (Greve, Borum & Pedersen, 2003; Pedersen et al., 2016; Kong et al., 2019; Rasmusson et al., 2020). However, a reduction in ROL in *H. ovalis* observed in the present study was not likely a result of enhanced respiratory oxygen consumption as warming was also found to inhibit net DIC release in the BG tissues. This phenomenon is more likely driven by limited oxygen supply from photosynthetic activity. Lower net oxygen evolution rates and DIC uptakes rates in the light were seen in AG tissues at 40 °C alongside a decrease in ETR. In addition, we cannot rule out the possibility that warming may have enhanced respiratory oxygen consumption in the light in AG tissues (Rasmusson et al., 2017; Rasmusson et al., 2020). It has been reported that the respiratory demand of aboveground tissues is significantly higher than that of the belowground tissues (Kong et al., 2019). Thus, it could play a significant role in the whole plant oxygen balance, particularly at high temperatures. Again, these findings highlight the interrelation between leaf and root metabolisms in *H. ovalis*.

Inhibition of photosynthesis by warming was also detected in *C. rotundata* measured as lower ETR and *T. hemprichii* measured as lower ETR, oxygen release and DIC uptake. These results agree with previous reports in *C. rotundata*, *T. hemprichii* and other tropical seagrasses (Collier & Waycott, 2014; Pedersen et al., 2016; George et al., 2020; Rasmusson et al., 2020; Rasmusson et al., 2021; Yucharoen et al., 2021). Nevertheless, in the present study, such photoinhibition did not affect gas exchange in the BG tissues of *C. rotundata* and *T. hemprichii*, pointing to the significance of efficient regulation of internal oxygen levels in these two seagrasses.

Increasing temperature decreases oxygen solubility and carbon dioxide availability (Beardall, Beer & Raven, 1998; Vaquer-Sunyer et al., 2012); thus, the possibility should not be excluded that difference in temperature (30 °C *vs* 40 °C) may in part contribute to changes in oxygen and DIC exchange observed in our study. Although measurement of oxygen content in plant tissues is needed to evaluate the impacts of warming on seagrass metabolisms accurately, our results suggest that heat stress can impose detrimental effects on the seagrass energy balance of the three seagrass species, thus potentially decreasing their primary production in the long term (Collier & Waycott, 2014; George et al., 2020). In addition, the low oxygen level in meristematic tissues has been proposed to be one of the main drivers for seagrass die-off (Greve, Borum & Pedersen, 2003; Borum et al., 2005; Borum et al., 2006). Here, *H. ovalis* may be more susceptible as their barrier for oxygen loss seemed weaker than that of other species. Their capacity to relieve hypoxic conditions in the rhizosphere was also compromised under a warming scenario. Nevertheless, Acclimation mechanisms such as metabolic adjustment (Danaraj et al., 2021; Zhang et al., 2021; Nguyen et al., 2021) and changes in morphological and anatomical traits (Han et al., 2017; Artika et al., 2020) have been reported and may allow seagrasses to cope with hypoxic and heat stress at moderate levels. Thus, the acclimation potential of tropical seagrass species considering complex environmental and biotic attributes, require further investigation.

## Conclusions

Our data show that seagrasses inhabiting the same area respond differently to root hypoxia and temperature, possibly due to their differences in anatomical and physiological attributes. *Halophila ovalis* is highly dependent on photosynthesis and appears to be the most sensitive species with the highest tendency of oxygen loss in hypoxic sediment. At the same time, its root oxidation capacity may be compromised under warming scenarios.

## Supplemental Information

10.7717/peerj.12899/supp-1 Supplemental Information 1 Raw data for plant measurements, net oxygen and dissolved inorganic carbon exchange rates and electron transport rates (Tables 1–3 and Figures 5 & 6).

10.7717/peerj.12899/supp-2 Supplemental Information 2 Summary of statistical analyses.

10.7717/peerj.12899/supp-3 Supplemental Information 3 Net oxygen and dissolved inorganic carbon (DIC) exchange rates in belowground (BG) and aboveground (AG) of seagrass-free chamber (negative control) exposed to contrasting oxygen levels in the belowground compartment and a combination of different light co.

## Additional Information and Declarations

### Field Study Permissions

The following information was supplied relating to field study approvals (*i.e*., approving body and any reference numbers): We had indeed collected seagrasses from the field (outside our institutes) but in Thailand you are required to obtain permit only when you collect materials in national park areas and our collection site was not in a national park. We do not need permit to proceed with our research nor funding in this case and thus, we do not have any document to provide.

### Data Availability

The following information was supplied regarding data availability: All the results of statistical analyses are available in the Supplemental Files.
