# Supplementary material for: Comparative study on anatomical traits and gas exchange responses due to belowground hypoxic stress and thermal stress in three tropical seagrasses
Source: PeerJ. 2022 Feb 9;10:e12899. doi: 10.7717/peerj.12899 (PMC8840093; doi:10.7717/peerj.12899)
Supplement: Supplemental Information 2 [file peerj-10-12899-s002.docx]

Table S1 Summary of One-Way ANOVAs of anatomical parameter of root, rhizome and leaf of seagrasses. Significant values (p < 0.05) are shown in bold

| **Parameter** | **Effect** | **SS** | **df** | **MS** | **F** | **p** |
| --- | --- | --- | --- | --- | --- | --- |
| Root thickness | Between species | 262974.93197 | 2 | 131487.46599 | 73.28373 | **0.000006** |
|  | Within species | 10765.34659 | 6 | 1794.22443 |  |  |
|  | Total | 273740.27857 | 8 |  |  |  |
| Root epidermal layer thickness | Between species | 214.62179 | 2 | 107.31089 | 17.87777 | **0.002967** |
|  | Within species | 36.01485 | 6 | 6.00247 |  |  |
|  | Total | 250.63664 | 8 |  |  |  |
| Total root cortex thickness | Between species | 59471.38103 | 2 | 29735.69051 | 59.88601 | **0.000011** |
|  | Within species | 2979.22905 | 6 | 496.53818 |  |  |
|  | Total | 62450.61009 | 8 |  |  |  |
| Outer root cortex thickness | Between species | 5308.83389 | 2 | 2654.4169 | 3.86051 | 0.083617 |
|  | Within species | 4125.49232 | 6 | 687.58205 |  |  |
|  | Total | 9434.32621 | 8 |  |  |  |
| Middle root cortex thickness | Between species | 1721.65932 | 2 | 860.82966 | 19.14246 | **0.002487** |
|  | Within species | 269.81785 | 6 | 44.969642 |  |  |
|  | Total | 1991.47718 | 8 |  |  |  |
| Inner root cortex thickness | Between species | 20183.06433 | 2 | 10091.53216 | 15.98719 | **0.003944** |
|  | Within species | 3787.35618 | 6 | 631.22602 |  |  |
|  | Total | 23970.42051 | 8 |  |  |  |
| Root air lacuna numbers | Between species | 896.88889 | 2 | 448.44445 | 155.23077 | **0.000000** |
|  | Within species | 17.33333 | 6 | 2.88889 |  |  |
|  | Total | 914.22222 | 8 |  |  |  |
| Rhizome thickness | Between species | 2.57560 | 2 | 1.28780 | 79.98354 | **0.000005** |
|  | Within species | 966048.06314 | 6 | 161008.01052 |  |  |
|  | Total | 2.67220 | 8 |  |  |  |
| Rhizome epidermal layer thickness | Between species | 205.97902 | 2 | 102.98951 | 3.231435 | 0.111583 |
|  | Within species | 191.22680 | 6 | 31.87113 |  |  |
|  | Total | 397.20582 | 8 |  |  |  |
| Total rhizome cortex thickness | Between species | 108077.62150 | 2 | 54038.81075 | 2.01587 | 0.213956 |
|  | Within species | 160840.00179 | 6 | 26806.66697 |  |  |
|  | Total | 268917.62330 | 8 |  |  |  |
| Outer rhizome cortex thickness | Between species | 44054.1897 | 2 | 22027.0948 | 8.62444 | **0.017189** |
|  | Within species | 15324.1977 | 6 | 2554.0330 |  |  |
|  | Total | 59378.3874 | 8 |  |  |  |
| Middle rhizome cortex thickness | Between species | 18049.74064 | 2 | 9024.8703 | 0.58764 | 0.584707 |
|  | Within species | 92147.35992 | 6 | 15357.8933 |  |  |
|  | Total | 110197.10056 | 8 |  |  |  |
| Inner rhizome cortex thickness | Between species | 3644.3057 | 2 | 1822.15285 | 4.44668 | 0.065385 |
|  | Within species | 2458.6679 | 6 | 409.777798 |  |  |
|  | Total | 6102.9736 | 8 |  |  |  |
| Rhizome air lacuna numbers | Between species | 18886.2222 | 2 | 1822.15285 | 39.0570 | **0.000363** |
|  | Within species | 1450.6667 | 6 | 409.77798 |  |  |
|  | Total | 20336.8889 | 8 |  |  |  |
| Rhizome stele diameter | Between species | 13117.42492 | 2 | 6558.71246 | 4.9526 | 0.053683 |
|  | Within species | 7945.81548 | 6 | 1324.30258 |  |  |
|  | Total | 21063.24039 | 8 |  |  |  |
| Ratio of stele diameter and cortex thickness | Between species | 0.00407 | 2 | 0.00203 | 1.42969 | 0.310631 |
|  | Within species | 0.00853 | 6 | 0.00142 |  |  |
|  | Total | 0.01260 | 8 |  |  |  |
| Rhizome porosity percentage | Between species | 0.00407 | 2 | 0.00203 | 1.42969 | 0.310631 |
|  | Within species | 0.00853 | 6 | 0.00142 |  |  |
|  | Total | 0.01260 | 8 |  |  |  |
| Lamina thickness | Between species | 37052.55063 | 2 | 18526.27531 | 130.97584 | **0.000011** |
|  | Within species | 848.68820 | 6 | 141.44803 |  |  |
|  | Total | 37901.2388 | 8 |  |  |  |
| Adaxially epidermal layer thickness | Between species | 113.38926 | 2 | 56.69463 | 9.65180 | **0.013333** |
|  | Within species | 35.24397 | 6 | 5.87399 |  |  |
|  | Total | 148.63323 | 8 |  |  |  |
| Abaxially epidermal layer thickness | Between species | 27.36580 | 2 | 13.68290 | 4.02017 | 0.078040 |
|  | Within species | 20.42135 | 6 | 3.40356 |  |  |
|  | Total | 47.42135 | 8 |  |  |  |
| Mesophyll thickness | Between species | 33729.10177 | 2 | 16864.55089 | 80.28461 | **0.000004** |
|  | Within species | 1260.35736 | 6 | 210.05956 |  |  |
|  | Total | 34989.45913 | 8 |  |  |  |
| Number of cell layer in mesophyll | Between species | 68.22223 | 2 | 34.11111 | 307.00000 | **0.000000** |
|  | Within species | 0.66667 | 6 | .11111 |  |  |
|  | Total | 68.88889 | 8 |  |  |  |
| Vascular bundle numbers | Between species | 220.222 | 2 | 110.11111 | 495.50000 | **0.000000** |
|  | Within species | 1.33333 | 6 | 0.22222 |  |  |
|  | Total | 221.55556 | 8 |  |  |  |
| Leaf air lacuna numbers | Between species | 220.222 | 2 | 110.11111 | 495.50000 | **0.000000** |
|  | Within species | 1.33333 | 6 | 0.22222 |  |  |
|  | Total | 221.55556 | 8 |  |  |  |
| Leaf porosity percentage | Between species | 5078.49269 | 2 | 2539.24634 | 140.95450 | **0.000000** |
|  | Within species | 108.08753 | 6 | 18.01459 |  |  |
|  | Total | 5186.58022 | 8 |  |  |  |

Table S2 Summary of Two-Way ANOVAs of root porosity of apical, middle, basal root zone and diameter of seagrasses. Significant values (p < 0.05) are shown in bold

|  | **Effect** | **SS** | **df** | **MS** | **F** | **p** |
| --- | --- | --- | --- | --- | --- | --- |
| Porosity | Species | 324.33634 | 2 | 162.16817 | 20.71787 | **0.000001** |
|  | Root zones | 2494.85189 | 2 | 1247.42595 | 159.36546 | **0.000000** |
|  | Species × root zones | 421.88881 | 4 | 105.47220 | 13.47465 | **0.000001** |
|  | Error | 281.78837 | 36 | 7.82745 |  |  |
|  | Total | 10967.92563 | 45 |  |  |  |
| Diameter | Species | 324900.46495 | 2 | 162450.23248 | 20.84517 | **0.000001** |
|  | Root zones | 42420.51812 | 2 | 21210.25906 | 2.72164 | 0.079299 |
|  | Species × root zones | 38108.35178 | 4 | 9527.08794 | 1.22249 | 0.318429 |
|  | Error | 280554.62108 | 36 |  |  |  |
|  | Total | 7864161.71612 | 45 |  |  |  |

Table S3. Summary of repeated ANOVAs of oxygen and dissolved inorganic carbon (DIC) exchange in belowground and aboveground tissues of seagrasses in responses to hypoxia and normoxia treatments. Significant values (p < 0.05) are shown in bold.

| 1. Oxygen release in belowground tissues | | | | | |
| --- | --- | --- | --- | --- | --- |
| **Effect** | **SS** | **df** | **MS** | **F** | **p** |
| Species | 18.11659 | 2 | 9.05829 | 27.35796 | **0.00000** |
| Error | 10.92639 | 33 | 0.33110 |  |  |
|  |  |  |  |  |  |
| Treatment | 5.05803 | 1 | 5.05803 | 35.36915 | **0.00000** |
| Treatment × Species | 4.48141 | 2 | 2.24070 | 15.66851 | **0.00001** |
| Error | 4.71922 | 33 | 0.14301 |  |  |
|  |  |  |  |  |  |

| 1. DIC release in belowground tissues | | | | | |
| --- | --- | --- | --- | --- | --- |
| **Effect** | **SS** | **df** | **MS** | **F** | **p** |
| Species | 878749 | 2 | 439374.6 | 13.66451 | **0.000048** |
| Error | 1061096 | 33 | 32154.4 |  |  |
|  |  |  |  |  |  |
| Treatment | 19531 | 1 | 19531.1 | 1.33335 | 0.256504 |
| Treatment × Species | 30772 | 2 | 15386.0 | 1.05037 | 0.361212 |
| Error | 483389 | 33 | 14648.2 |  |  |

| 1. Oxygen release in aboveground tissues |  | | | | |
| --- | --- | --- | --- | --- | --- |
| **Effect** | **SS** | **df** | **MS** | **F** | **p** |
| Species | 38.083 | 2 | 19.041 | 1.3243 | 0.279760 |
| Error | 474.495 | 33 | 14.379 |  |  |
|  |  |  |  |  |  |
| Treatment | 6.639 | 1 | 6.639 | 0.6231 | 0.435523 |
| Treatment × Species | 51.410 | 2 | 25.705 | 2.4128 | 0.105202 |
| Error | 351.572 | 33 | 10.654 |  |  |

| 1. DIC uptake in aboveground tissues |  | | | | |
| --- | --- | --- | --- | --- | --- |
| **Effect** | **SS** | **df** | **MS** | **F** | **p** |
| Species | 989524 | 2 | 494762 | 9.9871 | **0.000406** |
| Error | 1634823 | 33 | 49540 |  |  |
|  |  |  |  |  |  |
| Treatment | 73781 | 1 | 73781 | 2.8926 | 0.098391 |
| Treatment × Species | 432776 | 2 | 216388 | 8.4837 | **0.001065** |
| Error | 841709 | 33 | 25506 |  |  |

Table S4. Summary of different ANOVAs of oxygen and dissolved inorganic carbon (DIC) exchange in belowground and aboveground tissues of seagrasses to responses to thermal treatments. Significant values (p < 0.05) are shown in bold.

| 1. Oxygen release in belowground tissues(*Repeated ANOVA*) |  | | | | |
| --- | --- | --- | --- | --- | --- |
| **Effect** | **SS** | **df** | **MS** | **F** | **p** |
| Species | 145.1451 | 2 | 72.5725 | 34.99620 | **0.000000** |
| Temperature | 12.5522 | 1 | 12.5522 | 6.05296 | **0.017177** |
| Species x Temperature | 11.7575 | 2 | 5.8788 | 2.83488 | 0.067660 |
| Error | 109.9075 | 53 | 2.0737 |  |  |
|  |  |  |  |  |  |
| Light | 14.7271 | 1 | 14.7271 | 19.86564 | **0.000043** |
| Light x Species | 16.6508 | 2 | 8.3254 | 11.23028 | **0.000086** |
| Light x Temperature | 3.5004 | 1 | 3.5004 | 4.72173 | **0.034275** |
| Light x Species x Temperature | 2.8221 | 2 | 1.4111 | 1.90341 | 0.159110 |
| Error | 39.2907 | 53 | 0.7413 |  |  |
|  |  |  |  |  |  |

| 1. Dissolved inorganic carbon release in belowground tissues (*Repeated ANOVA*) |  | | | | |
| --- | --- | --- | --- | --- | --- |
| **Effect** | **SS** | **df** | **MS** | **F** | **p** |
| Species | 56626.5 | 2 | 28313.3 | 8.66683 | **0.000587** |
| Temperature | 81855.4 | 1 | 81855.4 | 25.05634 | **0.000007** |
| Species x Temperature | 69073.4 | 2 | 34536.7 | 10.57184 | **0.000148** |
| Error | 163342.8 | 50 | 3266.9 |  |  |
|  |  |  |  |  |  |
| Light | 7275.4 | 1 | 7275.4 | 4.12667 | **0.047545** |
| Light x Species | 22100.8 | 2 | 11050.4 | 6.26786 | **0.003724** |
| Light x Temperature | 1275.2 | 1 | 1275.2 | 0.72331 | 0.399115 |
| Light x Species x Temperature | 1399.9 | 2 | 699.9 | 0.39700 | 0.674431 |
| Error | 88151.2 | 50 | 1763.0 |  |  |

| C.1 Oxygen uptake in aboveground tissues in darkness (*Two-Way ANOVA*) |  | | | | |
| --- | --- | --- | --- | --- | --- |
| **Effect** | **SS** | **df** | **MS** | **F** | **p** |
| Species | 28.9901 | 2 | 14.4950 | 3.6239 | **0.033485** |
| Temperature | 0.0066 | 1 | 0.0066 | 0.0016 | 0.967782 |
| Species × Temperature | 26.0613 | 2 | 13.0306 | 3.2578 | **0.046300** |
| Error | 211.9895 | 53 | 3.9998 |  |  |

| C2. Oxygen release in aboveground tissues in the  light (*Two-way ANOVA*) |  | | | | |
| --- | --- | --- | --- | --- | --- |
| **Effect** | **SS** | **df** | **MS** | **F** | **p** |
| Species | 82.336 | 2 | 41.168 | 1.25410 | 0.293510 |
| Temperature | 291.392 | 1 | 291.392 | 8.87666 | **0.004321** |
| Species × Temperature | 160.212 | 2 | 80.106 | 2.44026 | 0.096693 |
| Error | 1772.643 | 54 | 32.827 |  |  |

| D1. Dissolved inorganic carbon release in aboveground tissues in darkness (*Two-Way ANOVA*) |  | | | | |
| --- | --- | --- | --- | --- | --- |
| **Effect** | **SS** | **df** | **MS** | **F** | **p** |
| Species | 92344.8 | 2 | 46172.4 | 5.75253 | **0.005643** |
| Temperature | 2337.0 | 1 | 2337.0 | 0.29116 | 0.591871 |
| Species × Temperature | 5598.5 | 2 | 2799.2 | 0.34875 | 0.707271 |
| Error | 401322.6 | 50 | 8026.5 |  |  |

| D2. Dissolved inorganic carbon uptake in aboveground tissues in the light (*Two-Way ANOVA*) |  | | | | |
| --- | --- | --- | --- | --- | --- |
| **Effect** | **SS** | **df** | **MS** | **F** | **p** |
| Species | 73729 | 2 | 36864 | 1.70105 | 0.192681 |
| Temperature | 169473 | 1 | 169473 | 7.82013 | **0.007268** |
| Species × Temperature | 69753 | 2 | 34876 | 1.60932 | 0.210014 |
| Error | 1105243 | 51 | 21671 |  |  |

Table S5. Summary of different ANOVAs of electron transport rate (ETR) in photosystem II of seagrasses to responses to the different oxygen and thermal treatments. Significant values (p < 0.05) are shown in bold.

| 1. Electron transport rate in photosystem II under hypoxia and normoxia treatments   (*Repeated ANOVA*) |  | | | | |
| --- | --- | --- | --- | --- | --- |
| **Effect** | **SS** | **df** | **MS** | **F** | **p** |
| Species | 13290.3 | 2 | 6645.2 | 34.177 | **0.000003** |
| Error | 2916.5 | 15 | 194.4 |  |  |
| Oxygen | 33.8 | 1 | 33.8 | 0.142 | 0.711115 |
| Oxygen × Species | 3311.9 | 2 | 1655.9 | 6.986 | **0.007174** |
| Error | 3555.4 | 15 | 237.0 |  |  |

| B. Electron transport rate in photosystem II under thermal treatments (*Two-Way ANOVA*) |  | | | | |
| --- | --- | --- | --- | --- | --- |
| **Effect** | **SS** | **df** | **MS** | **F** | **p** |
| Species | 16681.1 | 2 | 8340.5 | 37.839 | **0.000000** |
| Temperature | 18801.2 | 1 | 18801.2 | 85.296 | **0.000000** |
| Species × Temperature | 2691.5 | 2 | 1345.7 | 6.105 | **0.003692** |
| Error | 14548.0 | 66 | 220.4 |  |  |
