# Supplementary material for: Comparative study on anatomical traits and gas exchange responses due to belowground hypoxic stress and thermal stress in three tropical seagrasses
Source: PeerJ. 2022 Feb 9;10:e12899. doi: 10.7717/peerj.12899 (PMC8840093; doi:10.7717/peerj.12899)
Supplement: Supplemental Information 3 [file peerj-10-12899-s003.docx]

Supplementary Table S1 Net oxygen and dissolved inorganic carbon (DIC) exchange rates in belowground (BG) and aboveground (AG) of seagrass-free chamber (negative control) exposed to contrasting oxygen levels in the belowground compartment and a combination of different light conditions and temperatures (30 and 40 °C).

A. Net oxygen and DIC exchange rates in the negative control under the different oxygen level in the belowground chamber

| Chamber | Oxygen cond. | ΔpH | DIC release rate (umol h^-1^) | ΔDO  (mgL^-1^) | Oxygen uptake rates (mg h^-1^) |
| --- | --- | --- | --- | --- | --- |
| BG | Normoxia | 0.03±0.01 | 0.94±0.23 | 0.00±0.02 | 0.00±0.00 |
|  | Hypoxia | 0.04±0.01 | 1.56±0.55 | 0.01±0.04 | 0.00±0.00 |
| AG | Normoxia | 0.01±0.01 | -4.92±4.37 | 0.04±0.02 | 0.04±0.01 |
|  | Hypoxia | 0.01±0.01 | -4.74±3.97 | 0.05±0.03 | 0.04±0.03 |

BG: Belowground; AG: Aboveground

B. Net oxygen and DIC exchange rates in the negative control under the combination of different light condition and temperatures

| Light cond. | Chamber | Temp.  (°C) | pH change | DIC exchange rate  (umol h^-1^) | | DO change  (mgL^-1^) | DO exchange  (mg h^-1^) | |
| --- | --- | --- | --- | --- | --- | --- | --- | --- |
|  |  |  |  | release | uptake |  | release | uptake |
| Dark | BG | 30 | 0.01±0.01 | 0.44±0.22 | - | 0.36±0.13 | - | 0.02±0.01 |
|  |  | 40 | 0.01±0.01 | 0.23±0.74 | - | 0.15±0.05 | - | 0.01±0.00 |
|  | AG | 30 | 0.00±0.00 | 3.25+2.13 | - | 0.07±0.02 | - | 0.06±0.02 |
|  |  | 40 | 0.01±0.00 | 3.66±3.86 | - | 0.10±0.01 | - | 0.08±0.00 |
| Light | BG | 30 | 0.01+0.01 | 0.55±0.36 | - | 0.20+0.09 | - | 0.01+0.01 |
|  |  | 40 | 0.02±0.02 | 0.97±1.21 | - | 0.41±0.16 | - | 0.03±0.01 |
|  | AG | 30 | 0.01±0.00 | - | -6.33±2.01 | 0.03±0.03 | 0.02±0.03 | - |
|  |  | 40 | 0.01±0.01 | - | -5.75±5.60 | 0.06±0.05 | 0.05±0.04 | - |

BG: Belowground; AG: Aboveground
